# Exploring Active Ingredients, Beneficial Effects, and Potential Mechanism of *Allium tenuissimum* L. Flower for Treating T2DM Mice Based on Network Pharmacology and Gut Microbiota

**DOI:** 10.3390/nu14193980

**Published:** 2022-09-25

**Authors:** Shan-Shan Zhang, Yu-Fei Hou, Shao-Jing Liu, Sen Guo, Chi-Tang Ho, Nai-Sheng Bai

**Affiliations:** 1 Department of Pharmaceutical Engineering, College of Chemical Engineering, Northwest University, Xi’an 710069, China; 2 Department of Food Science, College of Food Science and Technology, Northwest University, Xi’an 710069, China; 3 Department of Medicinal Chemistry, College of Pharmacy, Xi’an Medical University, Xi’an 710021, China; 4 Department of Food Science, Rutgers University, New Brunswick, NJ 08901, USA

**Keywords:** *A. tenuissimum* flower flavonoids, network pharmacology, molecular docking, gut microbiota, type 2 diabetes mellitus

## Abstract

Forty compounds were isolated and characterized from *A. tenuissimum* flower. Among them, twelve flavonoids showed higher α−glucosidase inhibition activities in vitro than acarbose, especially kaempferol. The molecular docking results showed that the binding of kaempferol to α−glucosidase (GAA) could reduce the hydrolysis of substrates by GAA and reduce the glucose produced by hydrolysis, thus exhibiting α−glucosidase inhibition activities. The in vivo experiment results showed that flavonoids−rich *A. tenuissimum* flower could decrease blood glucose and reduce lipid accumulation. The protein expression levels of RAC−alpha serine/threonine−protein kinase (AKT1), peroxisome proliferator activated receptor gamma (PPARG), and prostaglandin G/H synthase 2 (PTGS2) in liver tissue were increased. In addition, the Firmicutes/Bacteroidetes (F/B) ratio was increased, the level of gut probiotics *Bifidobacterium* was increased, and the levels of *Enterobacteriaceae* and *Staphylococcus* were decreased. The carbohydrate metabolism, lipid metabolism, and other pathways related to type 2 diabetes mellitus were activated. This study indicating flavonoids−rich *A. tenuissimum* flower could improve glycolipid metabolic disorders and inflammation in diabetic mice by modulating the protein expression and gut microbiota.

## 1. Introduction

Diabetes mellitus (DM) is a complex chronic metabolic disease. Type 2 diabetes mellitus (T2DM) accounts for more than 90% of diabetic patients. Previous studies showed that T2DM is always accompanied by glucolipid metabolism disorders. Conventional therapies such as long−term usage of insulin injection and other oral medicines might have some side effects, such as gastrointestinal discomfort and hepatic metabolic burden [1]. Chinese herbs and their active ingredients are considered potential therapeutic materials for diabetes based on the safety of natural ingredients. It has been shown that *Pueraria lobate* (kudzu), *Portulaca oleracea*, *Folium mori*, *Radix scutellariae*, and other traditional Chinese herbs are widely used for treating diabetes [2,3]. Isoflavone−rich kudzu extract could decrease blood glucose levels in diabetic Wistar rats [4]. *Portulaca oleracea* flavonoids could significantly improve liver injury and promote insulin secretion and glucose uptake in diabetic mice [5]. Although the anti−diabetic effects of flavonoids are wildly reported in animal studies, however, most individual flavonoid ingredients are not successful in treating diabetes in clinical research. On the other hand, a previous study has shown that dietary flavonoids could reduce the risk of T2DM [6].

Network pharmacology is a cross−disciplinary product of traditional pharmacology, biochemistry, and systems biology [7,8]. Network pharmacology is conducted based on multiple online platforms and related visualization software. Multi−target analysis methods are increasingly used to predict the major active ingredients in herbal medicines and their potential targets and mechanisms of action [9]. Furthermore, molecular docking is an important method for drug design and screening, which could predict the binding mode and stability of ligand and target by calculating binding energy [10]. Combining network pharmacological analysis with molecular docking could be a useful method to explain the mechanism of interaction between active ingredients and their targets.

*A. tenuissimum* flower (*Allium*, Liliaceae) is wildly distributed in China. *A. tenuissimum* flower with a unique aroma is a vegetable, pickled product, and condiment. Our previous study has characterized and identified volatile compounds and confirmed the key aroma compounds of the *A. tenuissimum* flower [11]. Previous studies had also focused on the nutritional analysis, antioxidant and antibacterial activities, and the inhibitory effect on α−glucosidase of the ethanol extract of *A. tenuissimum* flower [12,13,14]. α−Glucosidase is the key enzyme of glucose metabolism, which can hydrolyze glycosides to produce glucose and play a key role in the modulation of blood glucose levels [15]. The in vitro α−glucosidase inhibitory activity assay is widely used to evaluate the potential activity of natural compounds including flavonoids and phenolics. Studies have demonstrated that α−glucosidase inhibitors have a positive effect on T2DM animal models [16,17,18]. Due to the containing of multiple hydroxyl and glycosidic functional groups, flavonoids are considered potential effective α−glucosidase inhibitors. Therefore, it has been suggested that flavonoids might have a therapeutic effect on diabetes mellitus [19,20,21]. In this research, we will focus on the flavonoids of *A. tenuissimum* flower, their anti−diabetic activity, and potential mechanisms based on network pharmacology analysis and high−fat diet feeding plus a streptozotocin−induced mice model.

Therefore, we aimed to (1) isolate and characterize the ingredients of *A. tenuissimum* flower, (2) use network pharmacology analysis and molecular docking to screen active ingredients and potential targets, (3) perform animal experiments to explore the anti−diabetic activity of *A. tenuissimum* flower and to verify the results of network pharmacology analysis and molecular docking, (4) explore the modulation effect of *A. tenuissimum* flower on the gut microbiota of diabetic mice.

## 2. Materials and Methods

### 2.1. Chemicals and Reagents

Methanol, ethyl acetate, *n*−butanol, dichloromethane, petroleum ether, and ethanol were of analytical grade and purchased from Fuyu Fine Chemical Co., Ltd. (Tianjin, China). α−Glucosidase (from yeast), phosphate−buffered saline (PBS), and streptozotocin (STZ) were purchased from Sigma−Aldrich (St Louis, MO, USA). Na_2_CO_3_, 4−nitrophenyl α−D−galactopyranoside (PNPG), acarbose, and TBST buffer were obtained from Aladdin Biochemical Technology Co., Ltd. (Shanghai, China). Bicinchoninic acid (BCA) protein assay kit was purchased from Nanjing Jiancheng Bioengineering Institute (Nanjing China). Pre−stained protein marker was obtained from New Cell & Molecular Biotech Co., Ltd. (Suzhou, China). RAC−alpha serine/threonine−protein kinase (AKT1), peroxisome proliferator−activated receptor gamma (PPARG), prostaglandin G/H synthase 2 (PTGS2), β−actin, and HRP−conjugated Affinipure Goat Anti−Mouse IgG (H + L) were purchased from Proteintech Group, Inc. (Rosement, PA, USA).

### 2.2. A. tenuissimum Flower Extract Preparation

The *A. tenuissimum* flower was collected from Xingtai City (Hebei Province, China) in August 2020. The dried *A. tenuissimum* flower (10 kg) was extracted with 60 L 75% ethanol/water three times (24 h/time). Approximately 2268 g crude residue (AF) was obtained after concentration and then dispersed in water. Petroleum ether was used for degreasing and decolorizing. Ethyl acetate and *n*−butanol were used for liquid−liquid extraction sequentially, yielding about 120.97 g of ethyl acetate extract and 514.85 g of *n*−butanol extract. Sample AFr (ca. 635.8 g) included the ethyl acetate and *n*−butanol parts.

### 2.3. Isolation, Characterization, and Quantitation

The *n*−butanol fraction was separated into five fractions from B1 to B5 by silica gel (100−200 mesh) column chromatography eluting with the MeOH−CH_2_Cl_2_ system (from 1:30 to 1:1). In the subsequent separation process, both silica gel and CHP−20P column chromatography were used for the separation, and the elution system for CHP−20P column chromatography was a methanol−water system (volume ratio from 0:1 to 1:0). Thirty−one compounds were isolated from the *n*−butanol part. The ethyl acetate fraction was separated into three fractions from EA1 to EA3 by column chromatography with silica gel (200−300 mesh) eluting with the MeOH−CH_2_Cl_2_ system from 1:20 to 1:1. The silica gel and CHP−20P column chromatography were used for further separation, and also the recrystallization method. Finally, nine compounds were obtained from the ethyl acetate part. The separation details were shown in Figure S1. The chemical structures of isolated compounds were characterized by comparing ^13^C NMR and ^1^H NMR data from the literature.

High performance liquid chromatography (HPLC) analysis was conducted by using an Agilent 1260 Infinity LC (Agilent Technologies, USA) equipped with a Phenomenex Gemini 5 μm C18 column (250 × 4.60 mm). The HPLC−DAD analysis conditions were as follows: the mobile phase was composed of 0.2% phosphoric acid aqueous solution (A) and methanol (B) with a gradient elution program: 0 min, 95% A; 10 min, 85% A; 20 min, 75% A; 35 min, 65% A; 40 min, 55% A; 50 min, 35% A; 60 min, 15% A; 70 min, 5% A. The flow rate was 1.0 mL/min, and the column temperature was 25 °C. The content of flavonoids from the extract of *A. tenuissimum* flower (AF) and AFr was detected through HPLC analysis at 254 nm wavelength using the external standard method (Figure S2).

### 2.4. Network Pharmacology Predictive Analysis

#### 2.4.1. Collecting and Analyzing Targets of Ingredients and T2DM

Active ingredients were screened in the Traditional Chinese Medicine Systems Pharmacology (TCMSP, https://tcmsp-e.com/tcmsp.php, accessed on 17 June 2022) database. Oral bioavailability (OB) and drug−likeness (DL) were the most important pharmacokinetic parameters of absorption, distribution, metabolism, and excretion (ADME). The active ingredients were screened by filter criteria OB ≥ 30% and DL ≥ 0.18. The compound information including Mol ID, structure, and relevant targets name was collected. Meantime, the disease−related targets were retrieved from a database of gene−disease associations DisGeNET platform (https://www.disgenet.org/, accessed on 17 June 2022) by using the “diabetes mellitus” term. The targets were normalized in the UniProt database (https://www.uniprot.org/, accessed on 19 June 2022) with “human species” and “reviewed” filter criteria. To find the common targets of active ingredients−T2DM, the screened active ingredients targets and T2DM targets were visualized in a draw Veen diagram online platform (http://bioinformatics.psb.ugent.be/webtools/Venn/, accessed on 20 June 2022).

#### 2.4.2. Protein−Protein Interaction Network Construction and Key Target Analysis

To explore the interaction of the active ingredients’ targets for treating T2DM, the common targets of active ingredients−T2DM were imported into an online platform STRING (version 11.5, https://cn.string-db.org/, accessed on 21 June 2022) to construct a protein−protein interaction (PPI) network. And the PPI network results were analyzed and visualized by Cytoscape v.3.9.0. The Centiscape 2.2 plug−in of Cytoscape was used to screen high interaction targets based on Closeness unDir threshold, Betweeness unDir threshold, and Degree unDir threshold. 

#### 2.4.3. GO and KEGG Enrichment Analysis

Gene ontology (GO) analysis and Kyoto encyclopedia of genes and genomes (KEGG) pathway enrichment analysis were conducted on the platform Metascape (http://metascape.org/gp/index.html#/main/step1, accessed on 21 June 2022). The main GO terms of potential targets were analyzed and the terms in biological process (BP), cellular component (CC), and molecular function (MF) were enriched. The KEGG pathways were analyzed and enriched. The significant enrichment terms (*p* ≤ 0.01) were selected to visualize through an online platform (http://www.bioinformatics.com.cn/, accessed on 21 June 2022).

#### 2.4.4. Ingredient-Target Protein Molecular Docking

According to the above−mentioned analysis, the key proteins were obtained. The 2D structures of active ingredients were downloaded from PubChem (https://pubchem.ncbi.nlm.nih.gov/, accessed on 22 June 2022), then converted to 3D structures in Chem3D software and saved as a docking ligand mol2 format. The 3D structures of key proteins were obtained from the PSCB PDB platform (https://www.rcsb.org/, accessed on 22 June 2022). The key proteins were removed from organic and solvent, and added hydrogen by using PyMOL software. The Autodock (v.4.2.6) software was used for molecular docking. Finally, the visualization of molecular docking results was performed by PyMOL software.

### 2.5. In Vitro α−Glucosidase Inhibitory Activity Assay

The α−glucosidase inhibitory activity assay method of *A. tenuissimum* flower flavonoids was slightly modified based on previous studies [22,23,24]. A 96−well plate was used for preparing the mixture consisting of 0.1 M PBS (pH 6.8), 1.0 mg/mL sample solution, and 2 U/mL α−glucosidase, which were incubated at 37 °C for 10 min. 20 μL PNPG solution (2.5 Mm, dissolved in PBS) was added to each well to start the reaction and incubated at 37 °C. Twenty minutes later, 80 μL Na_2_CO_3_ solution (0.2 mol/L) was added to each well to terminate the reaction. The absorbance was measured at 405 nm by Tecan Infinite M200 PRO (Tecan Trading Co. Ltd., Shanghai, China). Acarbose was used as a positive control. The inhibitory rate was calculated by the absorbance formula below:

Inhibition% = [1 − (A_a_ − A_b_) / A_c_] × 100%

*a*. 20 μL PNPG + 80 μL Na_2_CO_3_ + 20 μL PBS + 20 μL sample + 20 μL enzyme;

*b*. 20 μL PNPG + 80 μL Na_2_CO_3_ + 40 μL PBS +20 μL sample;

*c*. 20 μL PNPG + 80 μL Na_2_CO_3_ + 40 μL PBS + 20 μL enzyme.

### 2.6. In Vivo Animal Experiments

Animal experiments protocols and procedures following the guidelines established by the China Science Council were approved by the Laboratory Animal Center of Xi’an Jiaotong University Health Science Center (license number: SCXK 2018−001). 

**Figure 1:**
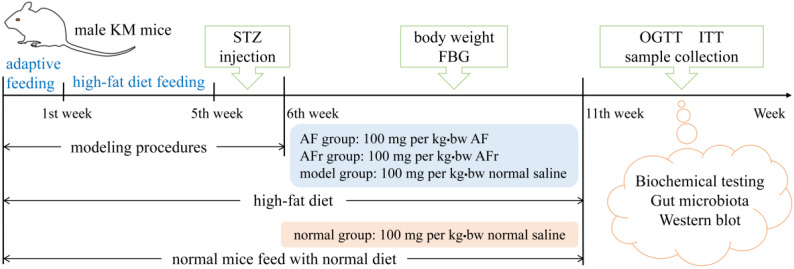
The schedule of animal experiment procedures. KM mice, Kunming mice; STZ, streptozotocin; FBG, fast blood glucose; OGTT, oral glucose tolerance test; ITT, insulin tolerance test. AF represents A. tenuissimum flower extract. AFr represents the mixture of ethyl acetate part and *n*-butanol part.

Sixty male KM (Kunming) mice (22 ± 2 g) were obtained from the laboratory animal center of Xi’an Jiaotong University Health Science Center. All mice were raised at a temperature of 25 ± 2 °C, relative humidity of 40−60%, and 12 h light/dark cycles laboratory conditions with free access to water and food. The schematic diagram of the animal experiments is shown in Figure 1. After adaptive feeding for one week, they were divided into two groups. The normal group (8 mice) was provided with a normal diet. The other group was provided with a high−fat diet (HFD) containing 45% energy from fat (FBSH Biotechnology Co., Ltd., Shanghai, China). After feeding for four weeks, the HFD−fed group mice were intraperitoneally injected with a dose of 35 mg per kg·bw 0.1 mol/L streptozotocin (STZ) dissolved in pH 4.5 Citric Acid−Sodium Citrate Buffer (Sigma−Aldrich, St Louis, MO, USA) for three consecutive days, while the normal group mice received the same dose of citric acid−sodium citrate buffer. 

The mice with fasting blood glucose (FBG) ≥ 11.1 mmol/L were considered diabetic mice and divided into three groups for treating 35 days [3]. The AF group mice were fed with 100 mg per kg·bw *A. tenuissimum* flower extract (AF) every day. The AFr group mice were fed with 100 mg per kg·bw the mixture of ethyl acetate part and *n*−butanol part (AFr) every day. The model group and the normal group were treated with the same dose of normal saline. Mice’s body weight and FBG were recorded once a week. After the last treatment, the mice fasted for 12 h to carry out an oral glucose tolerance test (OGTT) and insulin tolerance test (ITT). After the OGTT and ITT, the blood samples of all mice were collected at −80 °C. After euthanizing, liver and colon contents were collected and stored at −80 °C until analysis.

### 2.7. Western Blot Analysis

The protein of liver tissue was extracted and measured in concentration using bicinchoninic acid (BCA) protein assay kit for further western blot analysis. Protein samples were separated using sodium dodecyl sulfate−polyacrylamide gel electrophoresis (SDS−PAGE) and transferred to a 0.22 μm polyvinylidene fluoride (PVDF, Millipore, 6.6 × 8.5 cm) membrane. The membranes were blocked in 5% skimmed milk for 2 h at room temperature. Membranes were incubated with primary antibodies as follows overnight at 4 °C: anti−AKT1 (1:1000), anti−PTGS2 (1:1000), anti−PPARG (1:1000), and anti−β−actin (1:5000). After incubation, the membranes were washed three times and incubated at room temperature for 2 h with a secondary antibody goat anti−mouse IgG (1:5000). Then the membranes were washed three times with TBST buffer. Imaging protein bands were completed by using Tanon 5200 Multi (Tanon, Shanghai China), and ImageJ software was used for protein band grayscale analysis.

### 2.8. Gut Microbiota Analysis

The total DNA of colon samples was extracted according to the previous method [25]. PCR amplification of the V3−V4 region of the bacteria 16S rRNA genes was detected by 2% agarose gel electrophoresis and measured by fluorescence quantification. The Illumina Miseq platform was used to sequence. Alpha/beta diversity analysis, network analysis, species differences and marker species analysis, and functional forecasting were used to evaluate the diversity of the gut microbiota. 

### 2.9. Statistical Analysis

Origin 2017 (OriginLab, Northampton, Massachusetts, USA) used statistical analysis. The results were analyzed by GraphPad Prism 8 and expressed as mean ± standard deviation (mean ± SD). A value of *p* < 0.05 was considered significantly different.

## 3. Results and Discussion

### 3.1. Identification and Quantitation of Compounds in A. tenuissimum Flower

**Figure 2:**
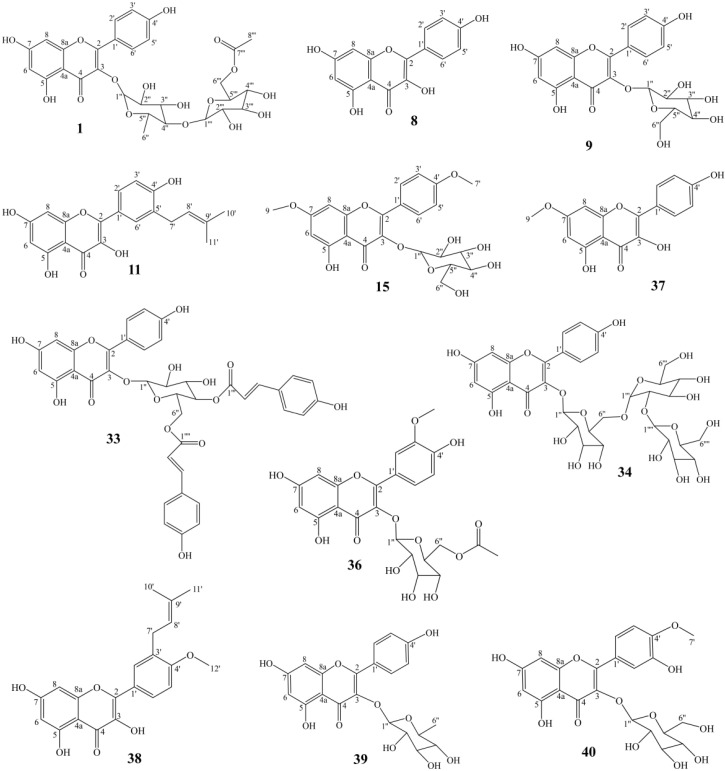
The structures of twelve flavonoids from *A. tenuissimum* flower.

A total of forty compounds numbering from AF−1 to AF−40 were obtained from *A. tenuissimum* flower. The chemical structures of these compounds were characterized by comparing their ^13^C NMR and ^1^H NMR data with those in the literature (Supplementary Materials). They are multiflorin A (AF−1), sonchifoliasolide G (AF−2), (-)−ent−prelacinan−7S−ol (AF−3), tritriacontane (AF−4), laricinolic acid (AF−5), di−D−fructose (AF−6), cedr−6−en−12−ol−14−oic acid (AF−7), kaempferol (AF−8), astragalin (AF−9), methyl 11,12,15−trihydroxy−13(14)−octadecenoate (AF−10), isolicoflavonol (AF−11), 8E−decaene-4,6−diyn−1−*O*−β−D−glucopyranoside (AF−12), methyl−α−D−fructofuranoside (AF−13), arbutin (AF−14), kaempferol−4’,7−dimethyl−3−*O*−glucoside (AF−15), 8*Z*−decaene−4,6− diyne−1−*O*−β−D−glucopyranoside (AF−16), (3*Z*) −3−hexene−1,5−diol 1−*O*−α−L−arabinopyranosyl (1→6) −β−D−glucopyranoside (AF−17), elentheroside E (AF−18), methyl−α−*D*−glucopyranoside (AF−19), burkholone (AF−20), gynostemoside C (AF−21), 5,7−dihydroxy−2−pentadecylchromen−4−one (AF−22), anisodamine (AF−23), onopornoid C (AF−24), 3−methylspongia−3,12−dien−16−one (AF−25), methyl 2,3,4,6−tetra−*O*−methyl−α−D−mannopyranosyl−(1→4)−6−*O*−acetyl−2,3−di−*O*−methyl−α−*D*−glucopyranoside (AF−26), β1−chaconine (AF−27), pestalafuranone E (AF−28), nortetillapyrone (AF−29), tricosanol (AF−30), pentacosane (AF−31), pinellactam (AF−32), kaempferol−3−*O*−β−*D*−4′′,6′′− di−(*E*)−p−coumaroyl glucoside (AF−33), kaempferol 3−*O*−β−*D*−glucopyranosyl−(1→2) −β−*D*−glucopyranosyl−(1→6)−β−*D*−glucopyranodide (AF−34), α−*D*−fructofuranose (AF−35), isorhamnetin 3−*O*−β−*D*−(6−acetyl)−galactopyranoside (AF−36), rhamnocitrin (AF−37), macasiamenol B (AF−38), afzelin (AF−39), tamarixin (AF−40). The chemical structures of all compounds were shown in Figure S3. Twelve flavonoids, eleven glycosides, five terpenes, and twelve other compounds were obtained from *A. tenuissimum* flower. The chemical structures of other flavonoids were derived from the skeleton of kaempferol (Figure 2). The quantitation of twelve flavonoids was developed by HPLC and the results are shown in Table 1. All calibration curves exhibited excellent linear regressions with the determination coefficients (R^2^) ranging from 0.9992 to 1.0000. The highest content was 284.1 μg/g (kaempferol, AF−8) and the lowest content was 15.1 μg/g (rhamnocitrin, AF−37) in the AFr sample. The total content of flavonoids was 1429.5 μg/g in the AFr sample. The highest content in the AF sample was 105.1 μg/g (multiflorin A, AF−1) and the lowest content was 7.6 μg/g (rhamnocitrin, AF−37). The total content of flavonoids was 402.0 μg/g in the AF sample. Total flavonoid content was higher in the AFr sample than that in the AF sample.

### 3.2. Network Pharmacology Analysis

#### 3.2.1. Potential Targets Analysis of Active Ingredients and T2DM

**Figure 3:**
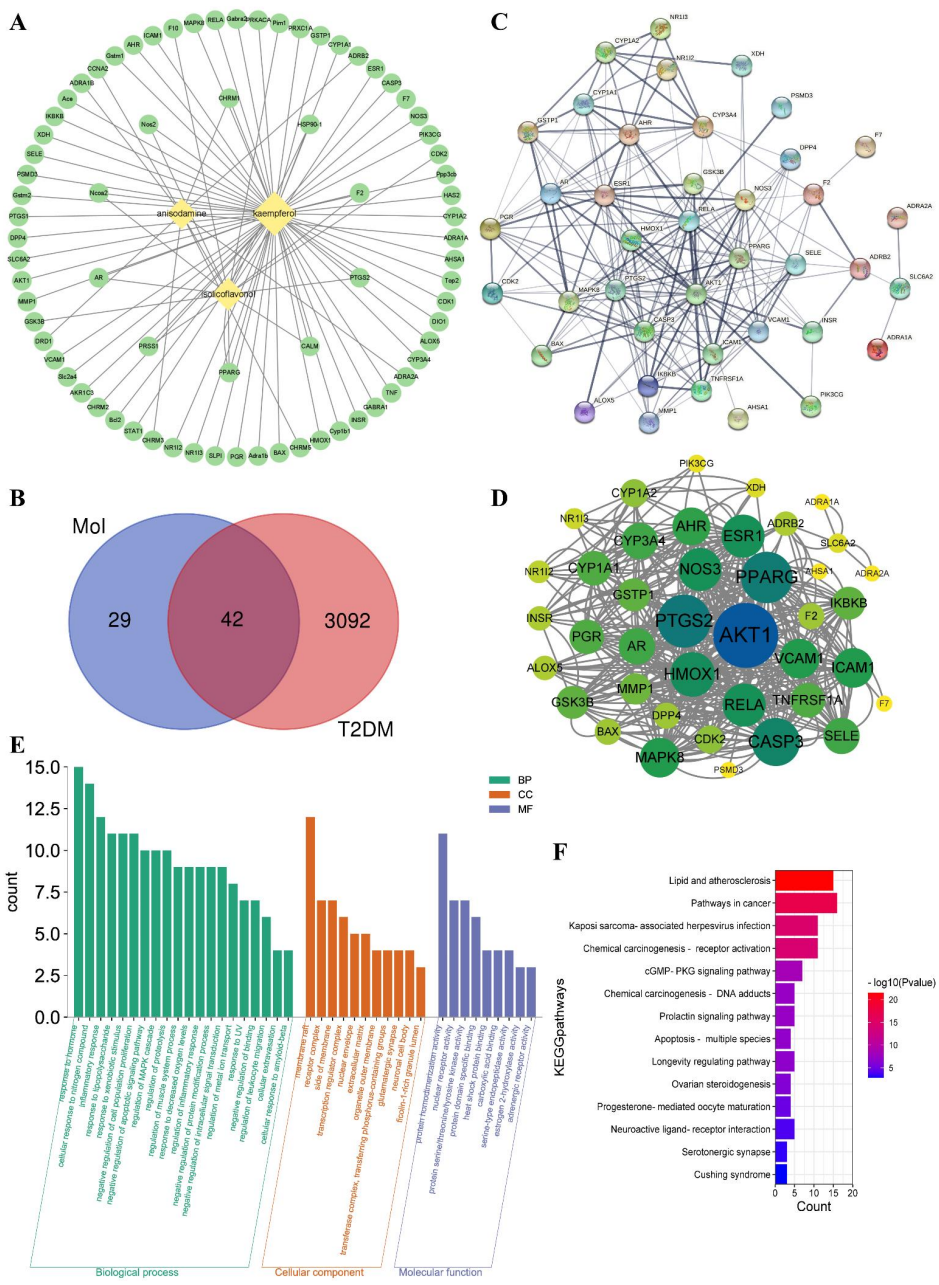
Network construction of ingredients−targets (**A**) and common targets Venn diagram of ingredients and T2DM (**B**), the “diamond” nodes represent the active ingredients. The “ellipse” nodes represent the potential targets. Protein−protein interaction (PPI) network analysis of potential targets of active ingredients−T2DM by STRING platform (**C**), and the high interaction modules analyzed by Centiscape 2.2 plug−in (**D**), the node size and color represented the connectivity degree. Gene ontology (GO) analysis (**E**) and Kyoto encyclopedia of genes and genomes (KEGG) pathway enrichment analysis (**F**) of active ingredients−T2DM potential targets.

Three active ingredients were collected as kaempferol (MOL000422), isolicoflavonol (MOL004949), and anisodamine (MOL005409) under OB ≥ 30% and DL ≥ 0.18 filter conditions, and the active ingredients−related targets were obtained from TCMSP database. The potential targets of T2DM were collected from the DisGeNET database. The “ingredients−targets” interaction network is shown in Figure 3A. A Venn diagram was used to analyze the ingredients’ targets and the disease’s targets. A total of 42 ingredient-diseases common targets were collected and used for further analysis (Figure 3B). Some information on those common targets is shown in Table S1.

#### 3.2.2. PPI Network Analysis

Protein−protein interaction (PPI) network analysis of ingredients−T2DM intersection targets was conducted by using the STRING platform (Figure 3C). The average node degree was 9.95 and the *p*−value was less than 1.0e-16. The STRING analysis results showed that AKT1, PPARG, PTGS2, and other proteins had high interaction. Then, the STRING analysis results were imported to Cytoscape software Centiscape 2.2 plug−in to analyze high interacting protein modules (Figure 3D). The Centiscape 2.2 analysis results showed the top three highly interacting targets were AKT1, PPARG, and PTGS2. The above results showed that AKT1 (RAC−alpha serine/threonine−protein kinase, PDB ID: 1UNQ), PPARG (peroxisome proliferator activated receptor gamma, PDB ID: 2PRG), and PTGS2 (Prostaglandin G/H synthase 2, PDB ID: 5F1A) might be good potential targets for molecular docking.

#### 3.2.3. GO Analysis and KEGG Pathway Enrichment

Metascape platform was used to analyze the 42 common targets of active ingredients−T2DM. The visualization results showed that the potential targets function related to biological processes (BP), cell components (CC), and molecular functions (MF). A total of 582 BP GO terms, 31 CC GO terms, and 56 MF GO terms were enriched, and the significantly enriched terms (*p* ≤ 0.01) were selected for analysis, respectively (Figure 3E). The results showed that the top 20 enriched BP GO terms involved response to lipopolysaccharide, negative regulation of apoptotic signaling pathway, inflammatory response, regulation of inflammatory response, and other biological processes−related terms. The significantly enriched CC GO terms included membrane raft, receptor complex, side of membrane, transcription regulator complex, and other cell components−related terms. The potential targets of MF GO terms were enriched in nuclear receptor activity, protein homodimerization activity, carboxylic acid binding, protein domain−specific binding, and other molecular function−related terms. For further potential mechanism exploring, the KEGG pathway enrichment analysis was conducted. The KEGG pathway enrichment results (Figure 3F) showed that fourteen pathways were enriched including lipid and atherosclerosis, pathways in cancer, and other related signaling pathways, suggesting the possible involvement and the potential mechanisms for *A. tenuissimum* flower in treating diabetes.

#### 3.2.4. Molecular Docking

**Figure 4:**
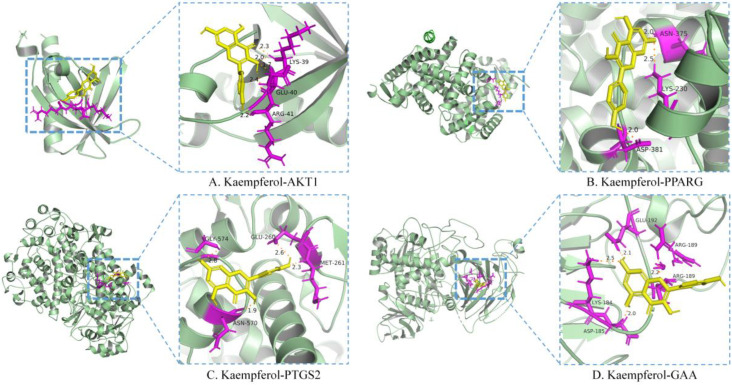
Molecular docking of the active ingredient and potential target by Autodock software.

Kaempferol was the only flavonoid of the active ingredients screened by network pharmacology analysis. Molecular docking was used to simulate the binding of kaempferol to the potential key targets AKT1, PPARG, and PTGS2. α−Glucosidase is a class of enzymes associated with glucose metabolism, and α−glucosidase (GAA, PDB ID: 5NN4) was also used as a receptor. Autodock (v.4.2.6) software was used to conduct molecular docking. The diagrams of ligand and potential target docking results are shown in Figure 4. It showed that kaempferol could bind to ARG−41, GLU−40, and LYS−39 residues of AKT1, the binding energy was −6.42 kcal·mol^−1^. The binding energy of kaempferol with GAA residues (ASP−185, LYS−184, GLU−192, and ARG−189) was −5.58 kcal·mol^−1^. The residues were binding sites for kaempferol on proteins. Detailed information on ligands and potential targets is listed in Table S2.

### 3.3. α−Glucosidase Inhibitory Activity of Flavonoids

**Figure 5:**
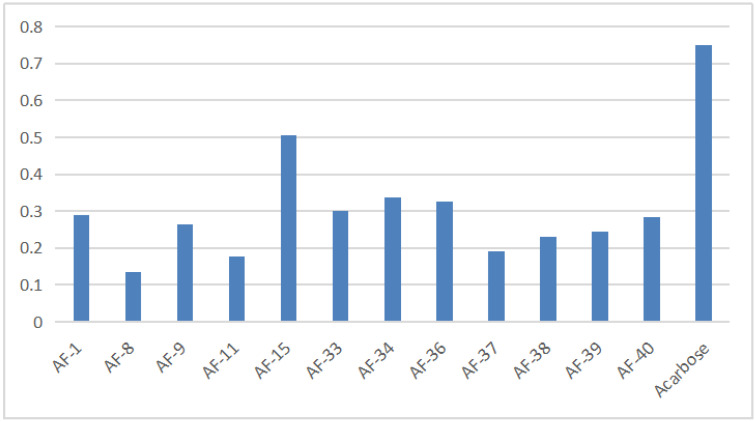
The result of *A. tenuissimum* flower flavonoids α−glucosidase inhibitory activity.

The results of the α−glucosidase inhibitory activity of flavonoids from *A. tenuissimum* flower were analyzed. It could be seen intuitively from the histogram in Figure 5 (More data listed in Table S3) that 12 flavonoids had superior α−glucosidase inhibitory activity than acarbose. Among them, AF−8 (kaempferol) showed superior α−glucosidase inhibitory activity (0.135 ± 0.011 mM) compared with acarbose (0.750 ± 0.002 mM). And the molecular docking results showed that kaempferol had high bonding energy with α−glucosidase (GAA), which was responsible for the high α−glucosidase inhibitory activity of kaempferol. The binding of kaempferol to GAA might replace the binding of substrate to GAA to exhibit the α−glucosidase inhibitory activity, which was consistent with the above experimental results. Other *A. tenuissimum* flower flavonoids were based on the kaempferol backbone with different substitution positions and substituents. Structurally, the C−ring phenolic hydroxyl hydrogen of AF−9 (astragalin) was substituted by the glucose group, and the experimental results showed that the α−glucosidase inhibitory activity of AF−8 was superior to that of AF−9 (0.264 ± 0.021 mM). The IC_50_ value of 0.506 ± 0.001 mM for compound AF−15 (kaempferol−4’,7−dimethyl−3−O−glucoside), in which the two hydroxyl hydrogens at the 4’ and 7 positions were substituted with methyl groups and the hydroxyl hydrogens at the 3 position was substituted with glucose groups, which had the smallest IC_50_ among the 12 flavonoids indicating that the α−glucosidase inhibitory activity of AF−15 was the lowest among these 12 flavonoids. The above results suggested that the reduction of phenolic hydroxyl groups in the B and C rings of the flavonoid backbone and the occurrence of glucosyl substitution led to the reduction of α−glucosidase inhibitory activity, which was consistent with the results of previous studies [24,26].

### 3.4. In Vivo Experiments Results

#### 3.4.1. Effect on Body Weight, FBG, OGTT, and ITT of Diabetic Mice

**Figure 6:**
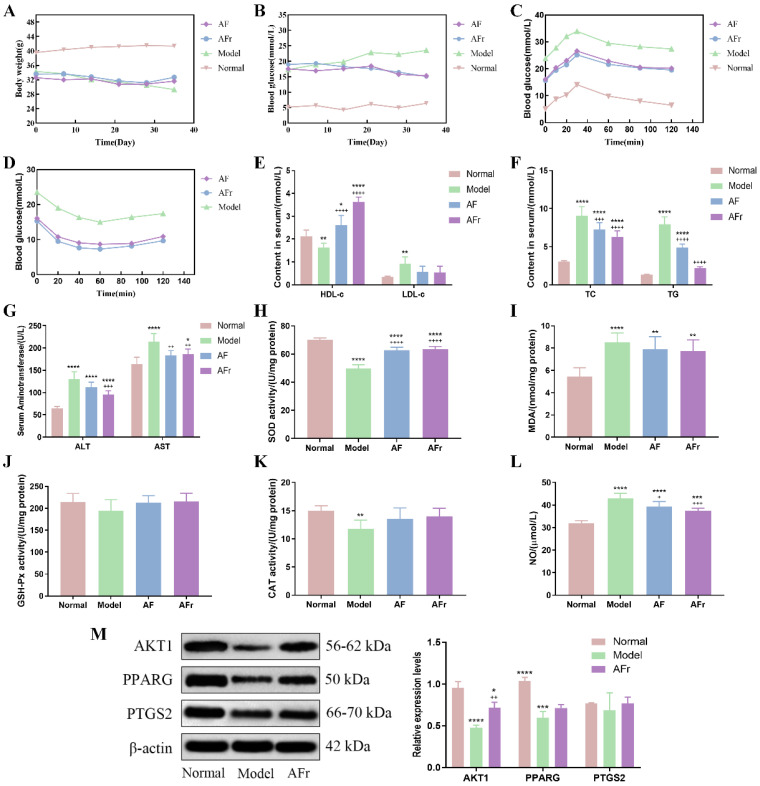
Effects on body weight (**A**), fasting blood glucose (FBG, **B**), oral glucose tolerance test (OGTT, **C**), insulin tolerance test (ITT, **D**), high/low−density lipoprotein cholesterol (HDL−c & LDL−c, **E**), total cholesterol & triglyceride (TC &TG, **F**), alanine aminotransferase & aspartate aminotransferase (ALT & AST, **G**), superoxide dismutase (SOD, **H**), malonaldehyde (MDA, **I**), glutathione peroxidase (GSH−Px, **J**), catalase (CAT, **K**), and nitric oxide (NO, **L**) (six mice per group). The expression levels of RAC−alpha serine/threonine−protein kinase (AKT1), peroxisome proliferator activated receptor gamma (PPARG), and prostaglandin G/H synthase 2 (PTGS2) proteins in liver tissue (**M**), data were shown as mean ± SD (n = 3). (compared to the normal group, * *p* < 0.05, ** *p* < 0.01, *** *p* < 0.005, **** *p* < 0.001; compared to the model group, + *p* < 0.05, ++ *p* < 0.01, +++ *p* < 0.005, ++++ *p* < 0.001).

The body weight, fasting blood glucose, OGTT, and ITT were measured and shown in Figure 6. After STZ injection, the body weight of the model group (34.32 ± 1.33 g) was lower than the normal group (39.57 ± 2.57 g). Compared with the normal group (5.2 ± 0.21 mmol·L^−1^), the FBG level was significantly increased in the model group (17.32 ± 0.46 mmol·L^−1^). The results showed that the T2DM model was successfully constructed. The OGTT and ITT were conducted after the treatment procedure. The results of OGTT showed that after 1.0 g per kg·bw glucose solution intragastrically, the blood glucose levels significantly increased in thirty minutes, and then gradually decreased. The peak serum glucose level of the model group was about 1.42 times the initial level. The peak blood glucose levels of the two treatment groups were about 1.60 times of initial levels. The ITT results showed a decreasing trend after injecting 1.0 U per kg·bw insulin solution, and the downward trends tended to be flat after 40 min. At 60 min after injection, the blood glucose in the AF group decreased by 46.0%, the blood glucose in the AFr group decreased by 52.2%, and the blood glucose in the Model group decreased by 36.2% compared to the initial values. The results showed that *A. tenuissimum* flower could decrease blood glucose, and improve diabetic mice’s blood glucose metabolism. And the AFr group with higher flavonoid content showed a better effect than the AF group.

#### 3.4.2. Effect on Serum Biomarkers of Diabetic Mice

Figure 6 showed that the insulin level of the model group was significantly decreased by 43.75% (*p* < 0.001) in the normal group while increased in the treatment groups. Compared with the normal group, the high−density lipoprotein cholesterol (HDL−c) level was decreased by 24.01% (*p* < 0.01) in the model group, and the HDL−c levels of two treatment groups were significantly increased especially in the AFr group (70.49%, *p* < 0.001). The low−density lipoprotein cholesterol (LDL−c) level of the model group was increased by 174.48% (*p* < 0.01) compared with the normal group. In the two treatment groups, the LDL−c levels were decreased. The total cholesterol (TC) and triglyceride (TG) levels of the model group were significantly increased than the normal group (*p* < 0.001). The TC and TG levels of the two treatment groups were decreased than the model group, but it still higher than the normal group. The alanine aminotransferase (ALT) and aspartate aminotransferase (AST) levels of the model group were significantly increased by 101.62% and 30.75% to the normal group, respectively. The levels of superoxide dismutase (SOD), catalase (CAT), and glutathione peroxidase (GSH−Px) were decreased in the model group compared with the normal group. After administration, the levels of SOD, CAT, and GSH−Px were increased in the AF group and the AFr group. The levels of malonaldehyde (MDA) and nitric oxide (NO) were significantly up−regulated (*p* < 0.001) in the model group and decreased after treatment. The histopathological results of the liver (Figure S4) showed that the normal group liver tissue had a clear structure and normal cell morphology. The model group’s liver tissue had many obvious fatty vacuoles, and the hepatocytes were enlarged and disordered. After treatment, the liver tissue injury degree was reduced, and the morphology tended to that of healthy mice. The above results showed that the AFr group with higher flavonoid content could better improve the abnormal lipid metabolism and ameliorate the oxidative stress and injury degree of the liver in diabetic mice. Flavonoid−rich *A. tenuissimum* flower could be a potential therapeutic material for diabetes.

#### 3.4.3. Western Blot Analysis

According to the above results, the AFr group with higher content of *A. tenuissimum* flower flavonoids showed an excellent anti−diabetic effect on mice. The protein expression levels in liver tissue were measured using Western blotting. All data were normalized by β−actin. As shown in Figure 6M, compared to the normal group, the expression levels of AKT1 (*p* < 0.001) and PPARG (*p* < 0.005) were significantly decreased in the model group. The expression level of PTGS2 was decreased without a significant difference. The expression level of AKT1 in the AFr group (*p* < 0.01) was up−regulated significantly compared with the model group. Also, the expression levels of PPARG and PTGS2 in the AFr group were up−regulated. The results indicated that the administration of AFr could increase the expression of these three proteins, especially AKT1 and PPARG. Previous studies showed that AKT1 might play a role in insulin−related pathways [27], and the increased expression of AKT1 plays a role in the treatment of T2DM and prevented its complications [28]. PPARG is a transcription factor revolving around adipocyte differentiation, lipid metabolism, and inflammation [29]. Some evidence has shown that PPARG is a candidate gene for obesity, insulin resistance, and T2DM [30,31,32]. PTGS2 has been confirmed to play a role in treating liver injury [33]. The KEGG pathways analysis indicated that the AKT1 participated in the TNF signaling pathway, lipid and atherosclerosis pathway, and regulation of lipolysis in the adipocytes pathway. The PPARG was the key protein of the PPAR signaling pathway, which revolved around adipocyte differentiation and lipid metabolism. The PTGS2 was related to the TNF signaling pathway, metabolism pathway, regulation of lipolysis in adipocytes, VEGF signaling pathway, and chemical carcinogenesis pathway. The results indicated that *A. tenuissimum* flower flavonoids could increase AKT1, PPARG, and PTGS2 expression levels, and play a role in T2DM by activating lipid metabolism and other related pathways.

#### 3.4.4. Gut Microbiota Analysis

**Figure 7:**
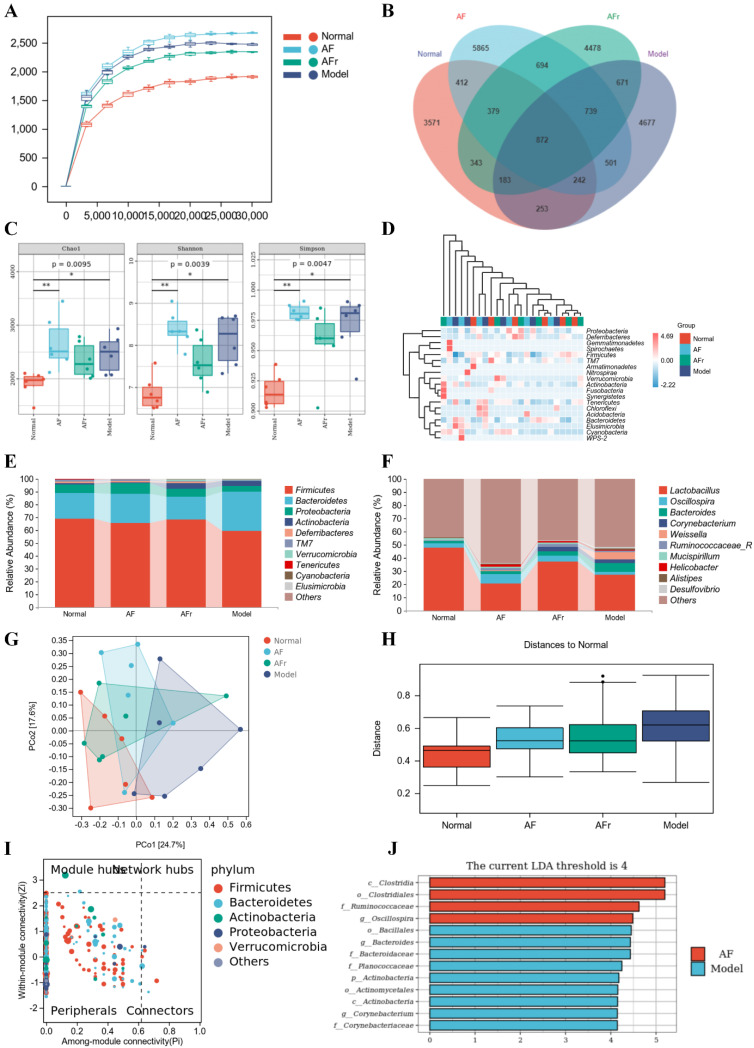
Effect of *A. tenuissimum* flower on gut microbiota consumption. Rarefaction curve (**A**), ASV/OTU Venn diagram (**B**), alpha diversity analysis (**C**, * *p* < 0.05, ** *p* < 0.01), the heat map of species composition at the phylum level (**D**), the gut microbiota abundance at the phylum level (**E**), and the gut microbiota abundance at the genus level (**F**), PCoA analysis (**G**), permutational multivariate analysis of variance (**H**), ZIPI−score diagram (**I**), and LEfSe analysis (**J**).

As shown in Figure 7A, the curves were gradually gentle as the sequencing depth increased, which reflected the diversity and abundance of samples. Figure 7B showed a total of 23,880 operational taxonomic units (OTUs) were detected from all samples. Venn diagram showed the number of unique or common species in four groups, and the differential species were the focus of subsequent research. The Chao 1 index, Shannon index, and Simpson index were used to reflect the results of alpha diversity analysis (Figure 7C). The levels of the Chao 1 index in the two treatment groups were higher than in the normal group. The increase of the Chao 1 index indicated that the species richness increased. The Shannon index and Simpson index reflected that the two treatment groups had higher species diversity than the normal mice. The results indicated that *A. tenuissimum* flower had a beneficial effect on improving the species richness and diversity of T2DM mice’s gut microbiota. The heat map (Figure 7D) visualized the differences and abundance of species composition. At the phylum level (Figure 7E), the species with higher relative abundance were *Firmicutes* (Normal: 68.97%, AF: 65.89%, AFr: 68.67%, Model: 59.44%), *Bacteroidetes* (Normal: 20.01%, AF: 22.63%, AFr: 17.45%, Model: 30.32%), *Proteobacteria*, and *Actinobacteria*, which together accounted for about over 95%. Compared with the normal group, the *Firmicutes* phylum was slightly lower, the *Bacteroidetes* phylum was higher, and the ratio of *Firmicutes*/*Bacteroidetes* (F/B) was lower in the model group. The F/B ratio in the AFr group (F/B = 3.93) was much higher than that in the model group (F/B = 1.96) and slightly higher than that in the normal group (F/B = 3.04) and the AF group (F/B = 2.91), which suggested that *A. tenuissimum* flower could improve the ratio of F/B in the intestine of diabetic mice and thus alleviate the symptoms of obesity. At the genus level (Figure 7F), the species with higher abundance were *Lactobacillus* (Normal: 48.07%, AF: 20.66%, AFr: 37.16%, Model: 27.63%), *Oscillospira*, *Bacteroides*, *Corynebacterium*, *Weissella*, *Ruminococcaceae*, and *Mucispirillum*. *Lactobacillus* had an important role in maintaining the health of the organism. Studies had shown that *Lactobacillus* could regulate gut microbiota, and reduce liver damage associated with T2DM [34]. It also had a regulatory effect on lipid metabolism, reduced high−fat diet−induced obesity in mice, as well as lowered blood glucose [35]. The relative abundance of *Lactobacillus* spp. was decreased in the model mice compared to normal mice. The relative abundance of *Lactobacillus* spp. in the AFr group was close to that of the normal group [36]. *Oscillospira* belongs to *Firmicutes*, which are widely found in the intestine of animals and humans and are positively associated with health [37]. 

To investigate the species differences among samples, beta diversity analysis was performed on all samples. Principal coordinates analysis (PCoA) and the between−group difference analysis (Figure 7G,H) were performed based on weighted unifrac. The results showed that the contribution of PCo1 was 24.7%, and PCo2 was 17.6%. The samples of the four groups were significantly different, especially between the normal group and the model group. The AFr group was closer to the normal group than the AF group. The between−group difference analysis results intuitively reflected the similarity and variability between samples. It could be seen that the gut microbiota of the normal group significantly differed from the model group, and the similarity of the gut microbiota between the two treatment groups was higher. And the distances between the two treatment groups and the normal group were closer than that between the treatment groups and the model group. This was consistent with the results of the PCoA analysis. 

As shown in Figure 7I, the key species at the phylum level were *Firmicutes*, *Bacteroidetes*, *Actinobacteria*, *Proteobacteria*, *Verrucomicrobia*, and this results were generally consistent with the results of species composition analysis. The results of the LEfSe analysis were shown in Figure 7J. When LDA values were equal to 4, a total of thirteen species with significant group differences (*p* < 0.5) in the AF group and the model group were found, while there had no significant differences between the AFr group and the normal group. Among them, four species in the AF group had significantly higher relative abundance than the model group, including *Clostridia*, *Clostridiales*, *Ruminococcaceae*, and *Oscillospira*. Most *Clostridia* are not pathogenic, and only a few *Clostridia* are pathogenic. *Ruminococcaceae* plays a crucial role in metabolism. *Ruminococcaceae* is one of the most effective bacteria to decompose carbohydrates and a key bacterium to degrade resistant starch, which can stabilize the intestinal barrier. *Oscillospira* belongs to the *Firmicutes*, which widely exists in animal and human intestines and is positively related to health [37].

**Figure 8:**
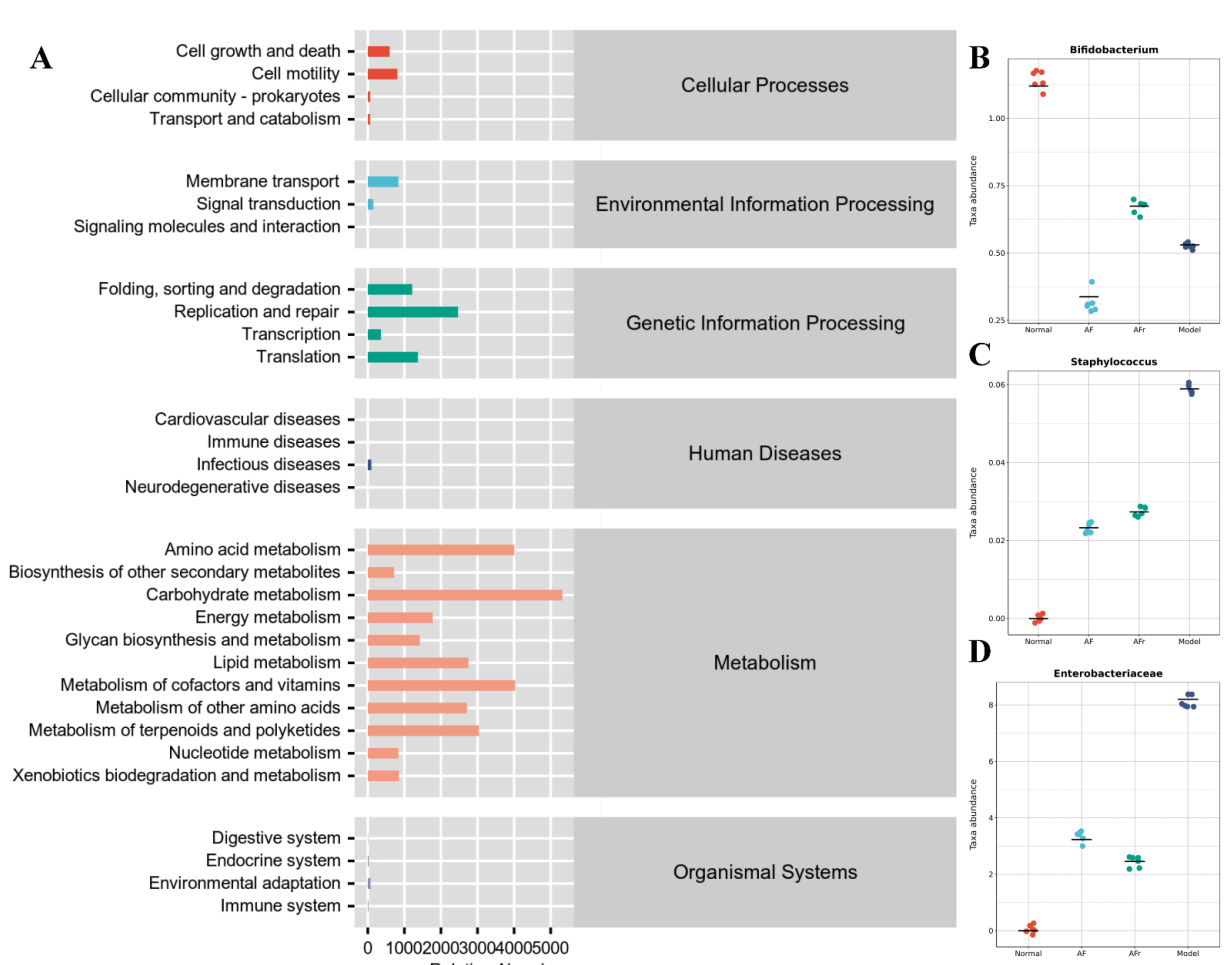
Metabolic pathways enrichment (**A**), and species composition difference analysis at the genus level between groups: (**B**) *Bifidobacterium*, (**C**) *Staphylococcus*, and (**D**) *Enterobacteriaceae*.

The KEGG pathway enrichment (Figure 8) results showed that many pathways had higher abundance, especially metabolism−related pathways. The KEGG pathways abundance heat map of all samples was shown in Figure S5. The metabolism−related pathways were arranged by abundance as carbohydrate metabolism, metabolism of cofactors and vitamins, amino acid metabolism, metabolism of terpenoids and polyketides, lipid metabolism, metabolism of other amino acids, energy metabolism, glycan biosynthesis and metabolism, nucleotide metabolism, xenobiotics biodegradation and metabolism, and biosynthesis of other secondary metabolites. Other pathways with high abundance include replication repair, translation, folding, sorting and degradation, membrane transport, cell motility, and other pathways. The results showed that the therapeutic effect of *A. tenuissimum* flower on T2DM was related to the modulation of metabolism-related pathways including carbohydrate metabolism, energy metabolism, lipid metabolism, and glycan biosynthesis and metabolism.

Species composition difference analysis at the genus level between groups was conducted based on the above pathways. *Enterobacteriaceae*, *Staphylococcus*, and *Bifidobacterium* had high abundance in the model group than in the other groups (Figure 8). *Staphylococcus* was a common purulent coccus that predisposes to a variety of purulent infections [38]. *Enterobacteriaceae* was usually found in fresh produce and many *Enterobacteriaceae* were known pathogens, e.g., *Salmonella* and pathogenic *E. coli* [39]. The microorganisms of the *Enterobacteriaceae* family induced inflammation and infection in vivo [40]. The results showed that the diabetic model mice had higher inflammation levels due to a significant increase of *Enterobacteriaceae* compared to the normal group. The *Bifidobacterium*, a probiotic with significant health benefits, was higher in the AFr group compared to the model group. *Bifidobacterium* could stimulate intestinal motility, purify the intestinal environment, stimulate the immune system, and improve immunity and anti−infection ability [41,42]. The experiment results showed that the abundance of *Enterobacteriaceae* and *Staphylococcus* microorganisms decreased, and the abundance of *Bifidobacterium* increased in the two treatment groups, indicating that *A. tenuissimum* flower could improve the inflammation in diabetic mice and enhance the anti-infection ability, thus alleviating the symptoms of diabetes.

## 4. Conclusions

To summarize, forty compounds were isolated and characterized by the *A. tenuissimum* flower. Twelve flavonoids were quantitatively analyzed as the main active ingredients of *A. tenuissimum* flower. Kaempferol had a higher content in *A. tenuissimum* flower compared with other flavonoids. Based on the network pharmacology analysis and molecular docking results, kaempferol could bind to AKT1, PPARG, PTGS2, and GAA. The expression levels of key target proteins AKT1, PPARG, and PTGS2 in liver tissue were increased in the AFr group by Western blot analysis. The flavonoids had higher α−glucosidase inhibitory activities than acarbose, especially kaempferol. The results of an animal experiment showed that *A. tenuissimum* flower could decrease blood glucose and lipid accumulation. The AFr group showed a better improvement effect in T2DM mice than the AF group. Based on quantitative analysis results, the total content of flavonoids in the AFr sample was higher than that of the AF sample. At the same dose, the AFr group mice were administrated with more flavonoids. That might be the reason for the AFr group showing an excellent alleviative effect on T2DM than that of the AF group. A dose of 100 mg per kg·bw of AF (AFr) was used on the T2DM mice model. The human equivalent dose (HED) for AF (AFr) was calculated as 8.1 mg/kg, which equates to a 486.5 mg dose of AF (AFr) for a 60 kg adult [43]. The plant *A. tenuissimum* flower is edible after being fried or pickled. The *A. tenuissimum* flower extract also could be made as a sauce and added to the daily diet as a condiment. Under the current dose, a consumption of *A. tenuissimum* flower might have potential health benefits for human and this data have some reference values for *A. tenuissimum* flower consumption. The gut microbiota analysis results showed that *A. tenuissimum* flower could modulate the species structure and abundance of diabetic mice, which might be related to the flavonoid content. The *A. tenuissimum* flower could modulate the ratio of F/B, decrease the level of *Enterobacteriaceae* and *Staphylococcus*, increase the level of *Bifidobacterium*, and activate carbohydrate metabolism, energy metabolism, lipid metabolism, glycan biosynthesis and metabolism, and other pathways related to T2DM and metabolism. It is indicated that *A. tenuissimum* flower could improve glycolipid metabolic disorders and inflammation in diabetic mice by modulating gut microbiota. *A. tenuissimum* flower is a healthy vegetable for daily consumption and also could be a potential medicinal ingredient. This research might provide support for subsequent applications of the *A. tenuissimum* flower, and will provide evidence for further study of the anti−diabetic mechanism of the *A. tenuissimum* flower and its active ingredients.

## Figures and Tables

**Table 1:** Quantitative analysis of twelve flavonoids in the AF and the AFr.

No.	Calibration Curves ^1^	R_2_	LOD ^2^ (μg/mL)	LOQ ^3^ (μg/mL)	Content in AFr (μg/g)	Content in AF (μg/g)
AF−1	y = 118.4x + 1441.9	0.9995	0.15	0.40	211.1	105.1
AF−8	y = 83.8x − 148.3	0.9999	0.05	0.25	284.1	59.2
AF−9	y = 185.1x − 177.3	0.9994	0.25	1.50	99.4	10.5
AF−11	y = 120.3x − 369.5	0.9997	0.50	1.25	188.8	26.8
AF−15	y = 279.6x − 226.0	0.9997	0.20	1.30	15.7	8.7
AF−33	y = 104.5x − 241.8	0.9998	0.15	0.70	196.3	54.7
AF−34	y = 352.5x − 292.2	0.9992	0.10	1.55	26.2	21.4
AF−36	y = 77.6x − 46.5	0.9997	0.45	2.75	143.7	37.3
AF−37	y = 57.2x + 121.7	0.9998	0.15	1.70	15.1	7.6
AF−38	y = 33.3x + 579.1	0.9998	0.20	1.50	171.6	21.1
AF−39	y = 304.6x + 280.8	1.0000	0.10	1.20	19.6	8.9
AF−40	y = 243.0x + 294.2	0.9995	0.05	0.50	57.9	40.7

^1^ y is the peak area, x is the concentration (μg/mL). ^2^ LOD is the detection limit which means the lowest detectable concentration. ^3^ LOQ is the quantification limit which means the lowest concentration that can be quantified. AF represents *A. tenuissimum* flower extract. AFr represents the mixture of ethyl acetate part and *n*−butanol part.

## Data Availability

All data generated or analyzed during this study are included in this published article (and its Supplementary Materials file).

## Supplementary Materials

The following supporting information can be downloaded at: https://www.mdpi.com/article/10.3390/nu14193980/s1, Figure S1. The separation process of *A. tenuissimum* flower; Figure S2. HPLC-DAD chromatograms of AF and AFr at 254 nm; Figure S3. The structure of forty compounds from *A. tenuissimum* flower; Figure S4. Histopathological results of H&E stained liver tissue; Figure S5. The heat map of the KEGG pathway abundance of all samples; Table S1. Common targets of active ingredients and T2DM; Table S2. Information of ligands and potential targets; Table S3. The α-glucosidase inhibitory activity of flavonoids from *A. tenuissimum* flower; NMR data [20,44,45,46,47,48,49,50,51,52,53,54,55,56,57,58,59,60,61,62,63,64,65,66,67,68,69,70,71,72,73,74,75,76,77,78,79,80,81,82].
