# Supplementary material for: Exploring Active Ingredients, Beneficial Effects, and Potential Mechanism of Allium tenuissimum L. Flower for Treating T2DM Mice Based on Network Pharmacology and Gut Microbiota
Source: Nutrients. 2022 Sep 25;14(19):3980. doi: 10.3390/nu14193980 (PMC9571170; doi:10.3390/nu14193980)
Supplement: Supplementary file 1 [file nutrients-14-03980-s001.zip › nutrients-1905421-supplementary.pdf]

nutrients

MDPI

Supplementary Materials

Exploring Active Ingredients, Beneficial Effects, and Potential Mechanism of *Allium tenuissimum* L. Flower for Treating T2DM Mice Based on Network Pharmacology and Gut Microbiota

Shan-Shan Zhang [1], Yu-Fei Hou [2], Shao-Jing Liu [3], Sen Guo [2], Chi-Tang Ho [4] and Nai-Sheng Bai [2,*]

Table of contents

Figure S1. The separation process of *A. tenuissimum* flower

Figure S2. HPLC–DAD chromatograms of AF and AFr at 254 nm

Figure S3. The structure of forty compounds from *A. tenuissimum* flower

Figure S4. Histopathological results of H&E stained liver tissue

Figure S5. The heat map of the KEGG pathway abundance of all samples

Table S1. Common targets of active ingredients and T2DM

Table S2. Information of ligands and potential targets

Table S3. The α–glucosidase inhibitory activity of flavonoids from *A. tenuissimum* flower

NMR data

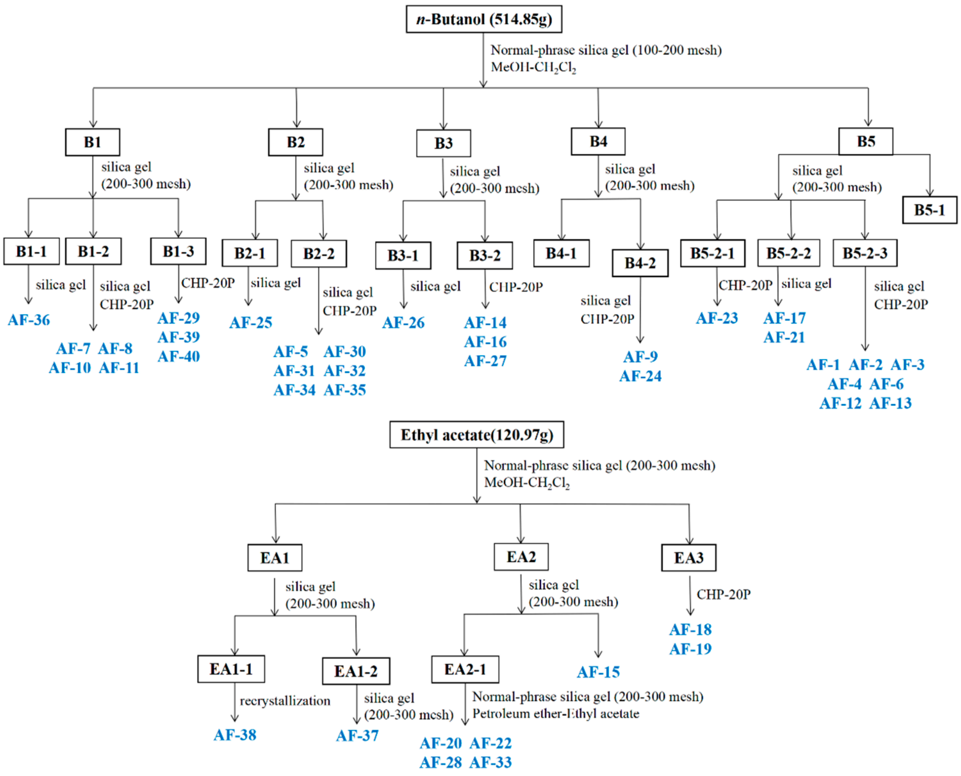

Figure S1. The separation process of *A. tenuissimum* flower.

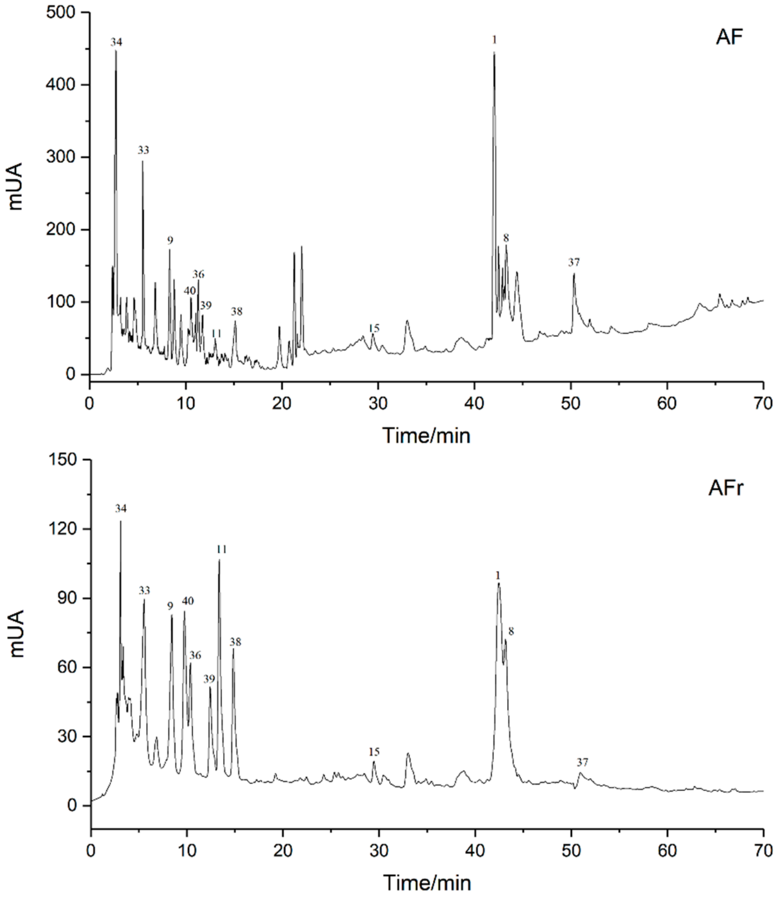

Figure S2. HPLC–DAD chromatograms of AF and AFr at 254 nm.

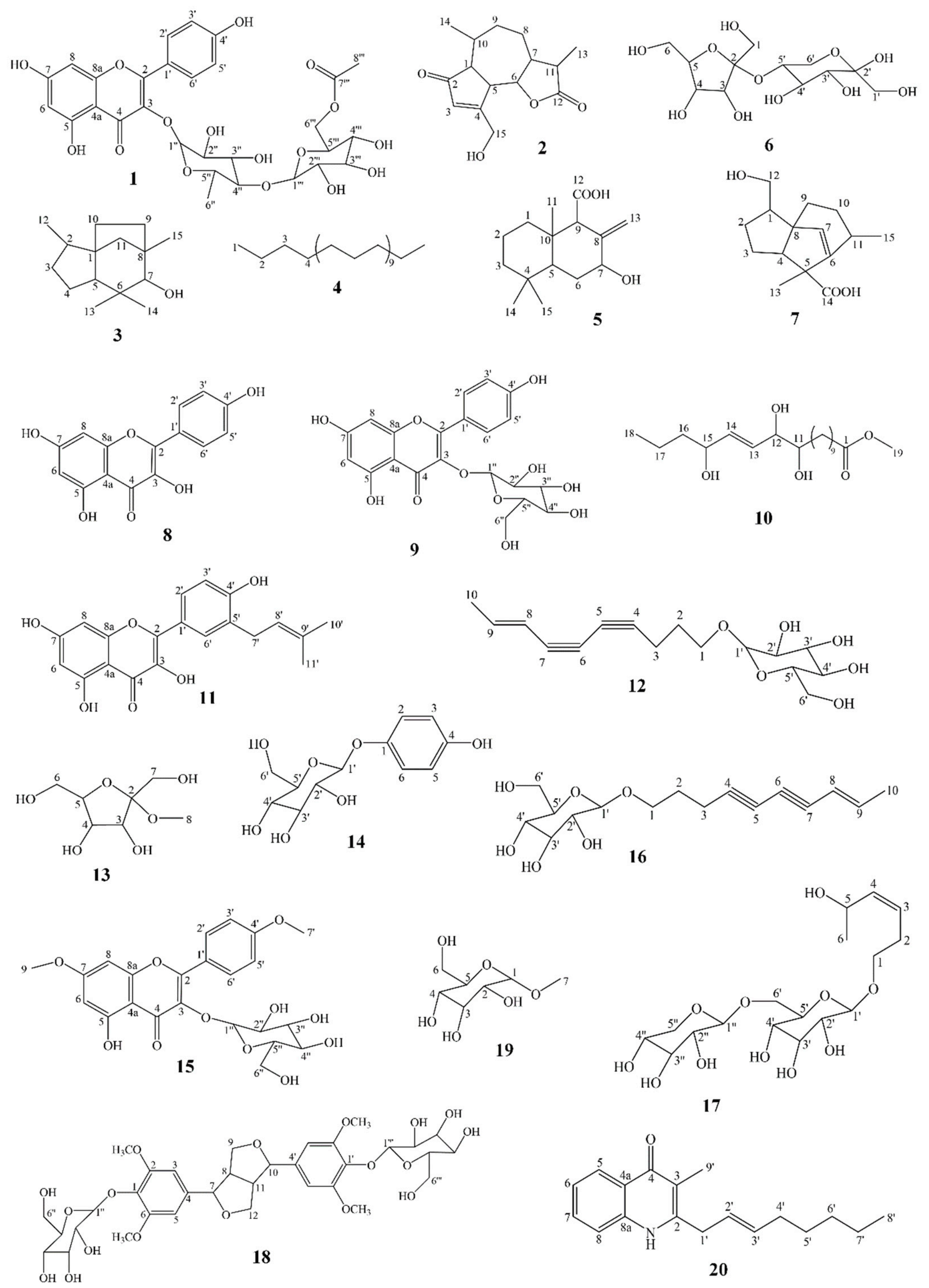
1
2
6
3
4
5
7
8
9
10
11
12
13
14
16
15
19
17
18
20

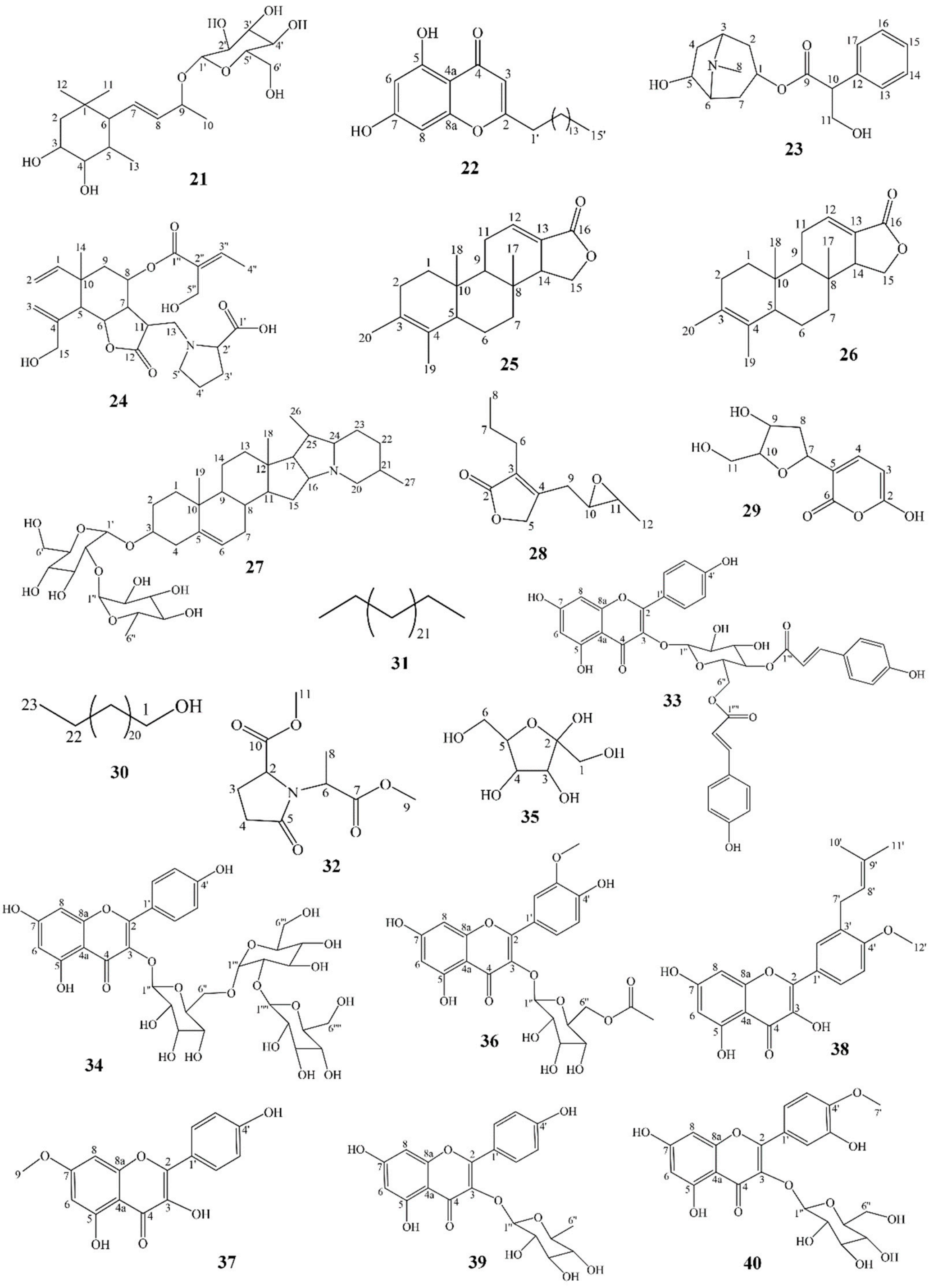

Figure S3. The structure of forty compounds from *A. tenuissimum* flower.

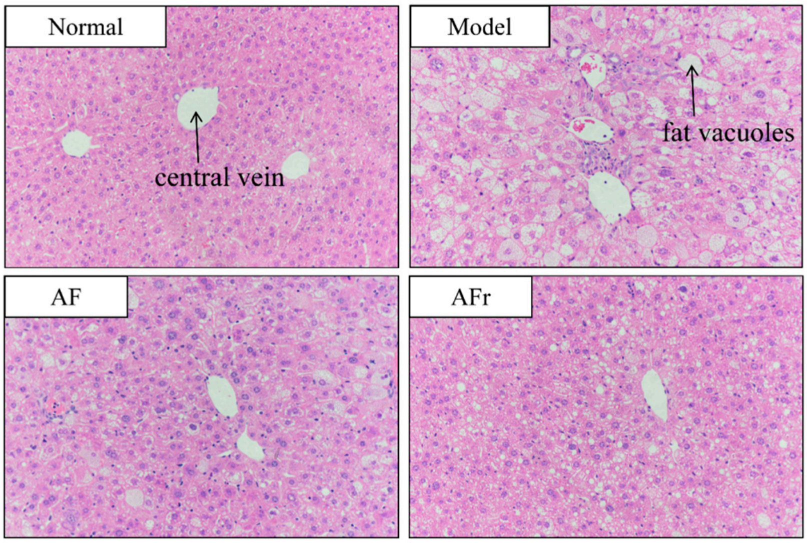

Figure S4. Histopathological results of H&E stained liver tissue.

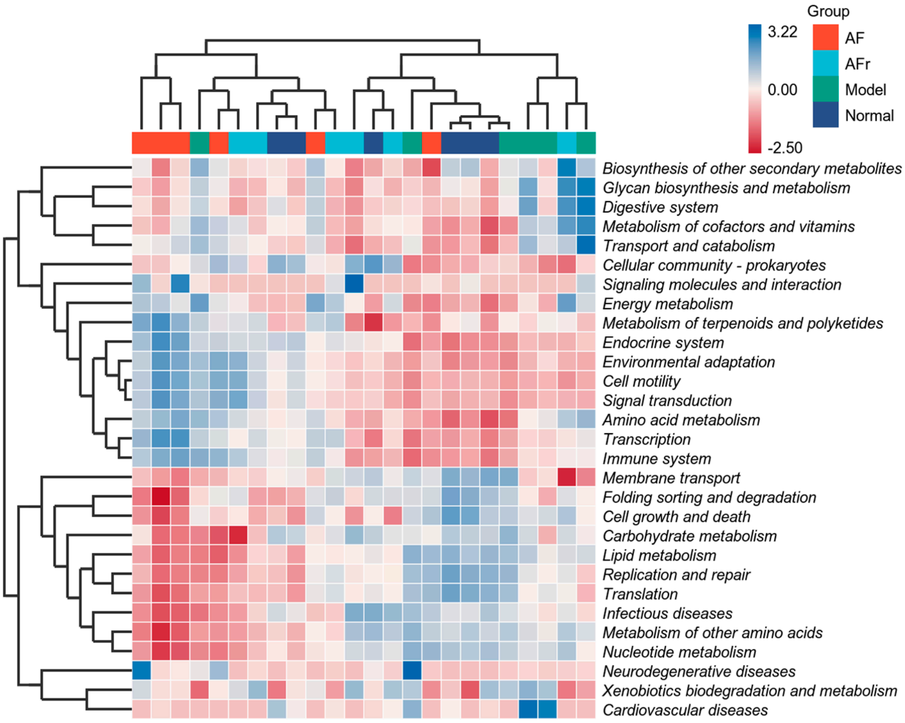

Figure S5. The heat map of the KEGG pathway abundance of all samples.

Table S1. Common targets of active ingredients and T2DM.

No.	Protein name	Uniprot ID	Gene symbol
1	Xanthine dehydrogenase/oxidase	P47989	XDH
2	26S proteasome non-ATPase regulatory subunit 3	O43242	PSMD3
3	adrenoceptor alpha 2A	P08913	ADRA2A
4	coagulation factor VII	P08709	F7
5	cytochrome P450 family 1 subfamily A member 1	P04798	CYP1A1
6	glutathione S-transferase pi 1	P09211	GSTP1

7	adrenoceptor alpha 1A	P35348	ADRA1A
8	aryl hydrocarbon receptor	P35869	AHR
9	nuclear receptor subfamily 1 group I member 3	Q14994	NR1I3
10	TNF receptor superfamily member 1A	P19438	TNFRSF1A
11	coagulation factor II, thrombin	P00734	F2
12	prostaglandin-endoperoxide synthase 2	P35354	PTGS2
13	estrogen receptor 1	P03372	ESR1
14	glycogen synthase kinase 3 beta	P49841	GSK3B
15	matrix metallopeptidase 1	P03956	MMP1
16	heme oxygenase 1	P09601	HMOX1
17	inhibitor of nuclear factor kappa B kinase subunit beta	O14920	IKBKB
18	mitogen-activated protein kinase 8	P45983	MAPK8
19	cyclin dependent kinase 2	P24941	CDK2
20	progesterone receptor	P06401	PGR
21	cholinergic receptor muscarinic 3	P20309	CHRM3
22	solute carrier family 6 member 2	P23975	SLC6A2
23	arachidonate 5-lipoxygenase	P09917	ALOX5
24	dipeptidyl peptidase 4	P27487	DPP4
25	peroxisome proliferator activated receptor gamma	P37231	PPARG
26	selectin E	P16581	SELE
27	secretory leukocyte peptidase inhibitor	P03973	SLPI
28	adrenoceptor beta 2	P07550	ADRB2
29	caspase 3	P42574	CASP3
30	cytochrome P450 family 1 subfamily A member 2	P05177	CYP1A2
31	activator of HSP90 ATPase activity 1	O95433	AHSA1
32	phosphatidylinositol-4,5-bisphosphate 3-kinase catalytic subunit gamma	P48736	PIK3CG
33	intercellular adhesion molecule 1	P05362	ICAM1
34	Transcription factor p65	Q04206	RELA
35	nitric oxide synthase 3	P29474	NOS3
36	vascular cell adhesion molecule 1	P19320	VCAM1
37	insulin receptor	P06213	INSR
38	AKT serine/threonine kinase 1	P31749	AKT1
39	nuclear receptor subfamily 1 group I member 2	O75469	NR1I2
40	Androgen receptor	P10275	AR
41	Apoptosis regulator BAX	Q07812	BAX
42	Cytochrome P450 3A4	P08684	CYP3A4

Table S2. Information of ligands and potential targets.

Ligands	Mol ID	Potential targets	PDB ID	Binding energy/(kcal·mol-1)
Kempferol	MOL000422	AKT1	1UNQ	-6.42
		PPARG	2PRG	-3.8
		PTGS2	5F1A	-3.96
		α-glucosidase (GAA)	5NN4	-5.58

Table S3. The α−glucosidase inhibitory activity of flavonoids from *A. tenuissimum* flower.

No.	Compound name	IC_{50}/mM(n=3)
AF−1	multiflorin A	0.289±0.014
AF−8	kaempferol	0.135±0.011
AF−9	astragalin	0.264±0.021
AF−11	isolicoflavonol	0.176±0.006
AF−15	kaempferol−4′,7−dimethyl−3−*O*−glucoside	0.506±0.001
AF−33	kaempferol−3−O−β−D−4′′,6′′ −di−(E)−*p*−coumaroyl glucoside	0.300±0.023
AF−34	kaempferol 3−O−β−D−glucopyranosyl− (1→2) −β−D−glucopyranosyl− (1→6) −β−D−glucopyranodide	0.335±0.001
AF−36	isorhamnetin 3−O−β−D− (6 −acetyl) −galactopyranoside	0.326±0.013
AF−37	rhamnocitrin	0.191±0.028
AF−38	macasiamenol B	0.230±0.034
AF−39	afzelin	0.244±0.032
AF−40	tamarixin	0.282±0.003
Acarbose		0.750±0.002

NMR data：

AF−1: multiflorin A[1]

^{13}C NMR (151 MHz, Methanol-*d*4) δ 157.7 (C-2), 134.7(C-3), 178.7(C-4), 162.3(C-5), 99.1 (C-6), 165.2 (C-7), 94.0 (C-8), 104.9 (C-4a), 158.3 (C-8a), 122.0 (C-1′), 131.5 (C-2′), 115.3 (C-3′), 160.8 (C-4′), 115.3 (C-5′), 131.5 (C-6′), 103.3 (C-1′′), 73.1 (C-2′′), 71.3 (C-3′′), 83.4 (C-4′′), 70.6 (C-5′′), 15.5 (C-6′′), 104.9 (C-1′′′), 77.2 (C-2′′′), 77.6 (C-3′′′), 71.3 (C-4′′′), 75.0 (C-5′′′), 61.9 (C-6′′′), 174.9 (C-7′′′), 20.7 (C-8′′′).

^{1}H NMR (600 MHz, Methanol-*d*4) δ 8.05 - 7.99 (m, 2H, H-2′, 6′), 6.87 (s, 2H, H-3′, 5′), 6.36 (s, 1H, H-8), 6.17 (s, 1H, H-6),5.23 (s, 1H, H-1′′), 4.53 - 4.47 (m, 1H, H-1′′′), 1.91 (s, 3H, H-8′′′), 0.93 - 0.84 (m, 3H, H-6′′).

AF−2: sonchifoliasolide G[2]

^{13}C NMR (151 MHz, Chloroform-*d*) δ 55.7 (C-1), 208.8 (C-2), 129.0 (C-3), 181.2 (C-4), 50.3 (C-5), 88.7 (C-6), 46.0 (C-7), 22.8 (C-8), 34.1 (C-9), 32.1 (C-10), 39.9 (C-11), 178.7 (C-12), 12.0 (C-13), 14.3 (C-14), 63.2 (C-15).

^{1}H NMR (600 MHz, Chloroform-*d*) δ 6.37 (d, 1H, J=2.0, H-3), 4.67 (dd, 2H, J=18.0, H-15), 4.50 (t, 1H, H-6), 3.22 (m, 5H, H-5), 2.77 (dd, 1H, H-1), 2.35 - 2.25 (m, 2H, H-7, 11), 2.06 - 1.95 (m, 1H, H-10), 1.67 - 1.57 (m, 4H, H-8, 9), 1.34 - 1.24 (m, 3H, H-13), 0.93 - 0.76 (m, 3H, H-14).

AF−3: (-)−ent−prelacinan−7S−ol[3]

^{13}C NMR (151 MHz, Chloroform-*d*) δ 53.4 (C-1), 39.3 (C-2), 32.2 (C-3), 21.3 (C-4), 58.2 (C-5), 38.1 (C-6), 86.1 (C-7), 45.3 (C-8), 30.0 (C-9), 23.0 (C-10), 50.4 (C-11), 14.4 (C-12), 16.4 (C-13), 34.3 (C-14), 25.1 (C-15).

^{1}H NMR (600 MHz, Chloroform-*d*) δ 3.43 - 3.35 (m, 1H, H-7), 2.29 (t, J = 7.6 Hz, 2H, H-9), 2.17 - 2.09 (m, 1H, H-2), 2.04 - 1.90 (m, 2H, H-3), 1.59 (dddd, J = 14.9, 9.8, 5.2, 2.4 Hz, 3H, H-4, 5), 1.45 - 1.34 (m, 2H, H-10), 1.34 - 1.24 (m, 2H, H-11), 1.24 (s, 3H, H-15), 1.22 (s, 3H, H-13), 1.10 - 1.05 (m, 3H, H-14), 0.78 (d, J = 6.9 Hz, 3H, H-12).

AF−4: tritriacontane[4]

^{13}C NMR (151 MHz, Chloroform-*d*) δ 14.2 (C-1), 22.8 (C-2), 32.0 (C-3), 29.8 (C-4).

^{1}H NMR (600 MHz, Chloroform-*d*) δ 1.29 - 1.25 (m, 62H, (CH2)31), 0.86 (t, J = 7.0 Hz, 6H, 2-Me).

AF−5: laricinolic acid[5]

^{13}C NMR (151 MHz, Chloroform-*d*) δ 41.2 (C-1), 29.7 (C-2), 41.3 (C-3), 32.0 (C-4), 54.9 (C-5), 32.0 (C-6), 72.6 (C-7), 147.2 (C-8), 62.8 (C-9), 38.9 (C-10), 14.2 (C-11), 173.0 (C-12), 105.4 (C-13), 22.7 (C-14), 19.9 (C-15).

^{1}H NMR (600 MHz, Chloroform-*d*) δ 5.26 (m, 1H, 7-OH), 5.04 (dd, J = 4.8, 0.8 Hz, 2H, H-13), 4.06 (td, J = 4.4, 2.6 Hz, 1H, H-7), 2.66 (s, 1H, H-9), 2.12 (ddd, J = 13.9, 6.8, 4.8 Hz, 4H, H-1, 3), 1.97 - 1.91 (m, 2H, H-2), 1.38 (s, 2H, H-6), 1.21 (s, 1H, H-5), 1.05 (m, 3H, H-11), 0.92 (s, 3H, H-14), 0.83 (t, J = 6.9 Hz, 3H, H-15).

AF−6: di−D−fructose[6]

^{13}C NMR (151 MHz, Methanol-*d*$_4$) δ 62.7 (C-1), 101.8 (C-2), 75.4 (C-3), 70.5 (C-4), 81.9 (C-5), 61.2 (C-6), 63.1 (C-1′), 97.9 (C-2′), 69.8 (C-3′), 68.1 (C-4′), 76.3 (C-5′), 64.5 (C-6′).

^{1}H NMR (600 MHz, Methanol-*d*$_4$) δ 4.60 (q, J = 7.7 Hz, 2H, H-1), 4.06 – 3.97 (m, 2H, H-1′), 3.85 (dd, J = 2.9, 1.7 Hz, 2H, H-3, 5′), 3.82 – 3.75 (m, 2H, H-6′), 3.75 – 3.68 (m, 1H, H-3′), 3.68 – 3.62 (m, 2H, H-4, 4′), 3.64 – 3.57 (m, 1H, H-5), 3.50 – 3.44 (m, 2H, H-6).

AF−7: cedr−6−en−12−ol−14−oic acid[7]

^{13}C NMR (151 MHz, Methanol-*d*$_4$) δ 29.4 (C-1), 29.1 (C-2), 22.4 (C-3), 33.6 (C-4), 34.1 (C-5), 131.0 (C-6), 128.6 (C-7), 51.8 (C-8), 28.9 (C-9), 28.7 (C-10), 31.7 (C-11), 65.3 (C-12), 24.8 (C-13), 176.4 (C-14), 13.1 (C-15).

^{1}H NMR (600 MHz, Methanol-*d*$_4$) δ 5.31 (tt, J = 6.3, 1.6 Hz, 1H, H-7), 4.26 - 3.85 (t, J = 4.2 Hz, 2H, H-12), 2.56 - 2.49 (m, 1H, H-11), 2.48 - 2.38 (m, 1H, H-4), 2.33 - 2.27 (m, 2H, H-2), 2.29 - 2.23 (m, 2H, H-3), 2.23 (s, 2H, H-9), 2.22 - 2.15 (m, 1H, H-1), 1.45 - 1.39 (m, 3H, H-13), 1.20 - 1.11 (m, 2H, H-10), 0.97 - 0.89 (m, 4H, H-15).

AF−8: kaempferol[8]

^{13}C NMR (151 MHz, Methanol-*d*$_4$) δ 146.7 (C-2), 135.8 (C-3), 176.0 (C-4), 103.2 (C-4a), 156.5 (C-5), 97.9 (C-6), 164.2 (C-7), 93.1 (C-8), 161.2 (C-8a), 122.4 (C-1′), 129.3 (C-2′), 115.0 (C-3′), 159.2 (C-4′), 115.0 (C-5′), 129.3 (C-6′).

^{1}H NMR (600 MHz, Methanol-*d*$_4$) δ 8.06 - 8.05 (m, 2H, H-2′, 6′), 6.87 - 6.86 (m, 2H, H-3′, 5′), 6.36 (d, J = 2.1 Hz, 1H, H-8), 6.15 (d, J = 2.1 Hz, 1H, H-6).

AF−9: astragalin[9]

^{13}C NMR (151 MHz, Methanol-*d*$_4$) δ 157.1 (C-2), 134.1 (C-3), 178.1 (C-4), 161.6 (C-5), 98.6 (C-6), 164.6 (C-7), 93.5 (C-8), 104.4 (C-4a), 157.8 (C-8a), 121.4 (C-1′), 131.0 (C-2′), 114.7 (C-3′), 160.2 (C-4′), 114.7 (C-5′), 131.0 (C-6′), 102.8 (C-1′′), 74.3 (C-2′′), 76.6 (C-3′′), 70.0 (C-4′′), 77.0 (C-5′′), 61.2 (C-6′′).

^{1}H NMR (600 MHz, Methanol-*d*$_4$) δ 8.04 - 7.99 (m, 2H, H-2′, 6′), 6.89 - 6.82 (m, 2H, H-3′, 5′), 6.34 (d, J = 2.0 Hz, 1H, H-8), 6.15 (d, J = 2.1 Hz, 1H, H-6), 5.20 (s, 1H, H-1′′), 3.91 (s, 2H, H-6′′), 3.66 (d, J = 2.4 Hz, 1H, H-3′′), 3.51 (dd, J = 11.9, 5.5 Hz, 1H, H-2′′), 3.48 - 3.38 (m, 2H, H-4′′, 5′′).

AF−10: methyl 11,12,15−trihydroxy−13(14) −octadecenoate[10]

13C NMR (151 MHz, Methanol-*d*$_4$) δ 174.7 (C-1), 22.4 (C-2), 24.7 (C-3), 25.1 (C-4), 25.3 (C-5), 28.8 (C-6), 29.0 (C-7), 29.2 (C-8), 31.8 (C-9), 32.2 (C-10), 71.7 (C-11), 74.5 (C-12), 129.7 (C-13), 135.2 (C-14), 75.2 (C-15), 33.5 (C-16), 37.0 (C-16), 13.1 (C-17), 50.6 (C-19).

1H NMR (600 MHz, Methanol-*d*$_4$) δ 5.70 - 5.64 (m, 1H, H-14), 5.47 - 5.40 (m, 1H, H-13), 4.04 - 4.00 (m, 1H, H-15), 3.90 - 3.84 (m, 1H, H-12), 3.78 - 3.68 (m, 3H, H-19), 3.34 - 3.26 (m, 1H, H-11), 2.28 (t, J = 7.4 Hz, 4H, H-2, 3), 1.57 (m, J = 7.4 Hz, 2H, H-16), 1.53 - 1.45 (m, 2H, H-10), 1.32 - 1.25 (m, 2H, H-17), 1.29 (s, 12H, H-4 ~ 9), 0.88 (td, J = 7.0, 2.1 Hz, 3H, H-18).

AF−11: isolicoflavonol[11]

13C NMR (151 MHz, Methanol-*d*4) δ 146.7 (C-2), 135.8 (C-3), 176.0 (C-4), 161.2 (C-5), 97.9 (C-6), 164.2 (C-7), 93.1 (C-8), 103.2 (C-4a), 156.9 (C-8a), 122.4 (C-1′), 129.3 (C-2′), 129.3 (C-3′), 159.2 (C-4′), 114.9 (C-5′), 129.3 (C-6′), 29.4 (C-7′), 122.4 (C-8′), 131.7 (C-9′), 28.4 (C-10′), 17.4 (C-11′).

1H NMR (600 MHz, Methanol-*d*4) δ 8.06 (d, J = 8.9 Hz, 1H, H-2), 7.85 (d, J = 8.8 Hz, 1H, H-8), 6.88 (d, J = 8.9 Hz, 2H, H-3, 6), 6.79 (d, J = 8.8 Hz, 1H, H-6′), 6.36 (d, J = 2.1 Hz, 3H, H-2′), 6.15 (d, J = 2.1 Hz, 1H, H-3′), 3.64 (s, 2H, H-7′), 1.28 (s, 3H, H-10′), 1.25 (s, 3H, H-11′).

AF−12: 8E−decaene−4,6−diyn−1−O−β−D−glucopyranoside[12]

13C NMR (151 MHz, Methanol-*d*4) δ 68.4 (C-1), 29.4 (C-2), 16.2 (C-3), 66.5 (C-4), 71.0 (C-5), 73.7 (C-6), 80.8 (C-7), 109.3 (C-8), 142.3 (C-9), 18.9 (C-10), 103.5 (C-1′), 72.5 (C-2′), 76.7 (C-3′), 70.0 (C-4′), 74.9 (C-5′), 61.5 (C-6′).

1H NMR (600 MHz, Methanol-*d*4) δ 7.70 (dd, 1H, H-9), 7.59 (dd, 1H, H-8), 4.51 - 4.43 (m, 1H, H-1′), 4.29 - 4.20 (m, 1H, H-2′), 3.89 - 3.82 (m, 1H, H-3′), 3.85 - 3.77 (m, 2H, H-4′, 5′), 3.66 - 3.60 (m, 2H, H-6′), 3.63 - 3.57 (m, 2H, H-1), 3.57 - 3.47 (m, 2H, H-2), 1.75 - 1.68 (m, 2H, H-3), 1.71 - 1.62 (m, 3H, H-10).

AF−13: methyl−*α*−D−fructofuranoside[13]

13C NMR (151 MHz, Deuterium Oxide) δ 108.3 (C-2), 80.1 (C-3), 77.4 (C-4), 83.3 (C-5), 61.3 (C-6), 57.7 (C-7), 48.2 (C-8)

1H NMR (600 MHz, Deuterium Oxide) δ 3.95 (d, J = 2.8 Hz, 1H, H-3), 3.85 - 3.79 (m, 2H, H-4,5), 3.72 - 3.61 (m, 2H, H-6), 3.61 - 3.46 (m, 2H, H-7), 3.18 (dd, J = 15.3, 4.3 Hz, 3H, H-8).

AF−14: arbutin[14]

13C NMR (151 MHz, Deuterium Oxide) δ 159.8 (C-1), 115.5 (C-2), 117.5 (C-3), 156.7 (C-4), 117.5 (C-5), 115.5 (C-6), 102.5 (C-1′), 73.1 (C-2′), 75.4 (C-3′), 69.4 (C-4′), 75.9 (C-5′), 60.6 (C-6′).

1H NMR (600 MHz, Deuterium Oxide) δ 7.52 (s, 2H, H-3, 5), 7.38 (s, 2H, H-2, 6), 6.47 (d, J = 9.1 Hz, 1H, H-1′), 3.73 (d, J = 12.6 Hz, 1H, H-6′), 3.20-3.66 (m, 4H, H-2′~5′).

AF−15: kaempferol−4',7−dimethyl−3−O−glucoside[15]

13C NMR (151 MHz, Methanol-*d*4) δ 157.7 (C-2), 134.1 (C-3), 178.2 (C-4), 161.7 (C-5), 98.5 (C-6), 164.6 (C-7), 93.4 (C-8), 104.4 (C-4a), 157.2 (C-8a), 56.0 (C-9), 121.4 (C-1′), 130.9 (C-2′), 114.7 (C-3′), 160.2 (C-4′), 114.7 (C-5′), 130.9 (C-6′).

1H NMR (600 MHz, Methanol-*d*4) δ 8.05 - 8.00 (m, 2H, H-2′, 6′), 6.88 - 6.84 (m, 2H, H-3′, 5′), 6.79 - 6.75 (m, 1H, H-6), 6.37 (d, J = 2.0 Hz, 1H, H-8), 6.17 (d, J = 2.1 Hz, 1H, H-1′′), 3.92 (s, 6H, H-9, 7′), 3.79 - 3.00 (m, 6H, H-2′′~6′′).

AF−16: 8Z−decaene−4,6−diyne−1−O−β−D−glucopyranoside[16]

13C NMR (151 MHz, Methanol-*d*4) δ 68.4 (C-1), 29.4 (C-2), 16.1 (C-3), 80.9 (C-4), 66.5 (C-5), 79.6 (C-6), 72.5 (C-7), 109.4 (C-8), 141.4 (C-9), 16.6 (C-10), 103.3 (C-1′), 73.7 (C-2′), 76.8 (C-3′), 70.0 (C-4′), 76.5 (C-5′), 61.1 (C-6′).

1H NMR (600 MHz, Methanol-*d*4) δ 6.37 - 6.39 (d, 1H, H-9), 5.47 (s, 1H, H-8), 4.27 - 4.23 (m, 1H, H-1′), 4.13 - 4.03 (m, 2H, H-1), 3.89 - 3.83 (m, 2H, H-6′), 3.41 (m, 3H, H-3′, 4′, 5′), 3.19 (d, J = 11.1 Hz, 1H, H-2′), 2.52 (s, 2H, H-3), 1.93 - 1.82 (m, 5H, H-2, 10).

AF−17: (3Z)−3−hexene−1,5−diol 1−O−*α*−L−arabinopyranosyl(1→ 6) −β−D−glucopyranoside [17]

13C NMR (151 MHz, Deuterium Oxide) δ 69.6 (C-1), 29.6 (C-2), 127.4 (C-3), 137.8 (C-4), 63.8 (C-5), 23.3 (C-6), 95.9 (C-1′), 75.7 (C-2′), 81.3 (C-3′), 71.1 (C-4′), 75.9 (C-5′), 69.1 (C-6′), 98.1 (C-1′′), 72.4 (C-2′′), 74.1 (C-3′′), 67.5 (C-4′′), 66.6 (C-5′′).

1H NMR (600 MHz, Deuterium Oxide) δ 5.22 (d, 1H, H-4), 5.04 (d, 1H, H-3), 4.22 (m, 1H, H-5), 4.28 (d, J = 6.5 Hz, 1H, H-1′′), 4.04 (d, J = 7.5 Hz, 1H, H-1′), 3.88 - 3.79 (dd, J = 5.5, 11.0 Hz, 3H, H-6′, 4′′), 3.76 - 3.68 (m, 2H, H-1), 3.68 - 3.40 (m, 4H, H-2′′, 3′′, 5′′), 3.39 - 3.34 (m, 1H, H-5′), 3.31 - 3.25 (m, 2H, H-3′, 4′), 3.15 (d, J = 7.5 Hz, 1H, H-2′), 2.22 - 2.32 (m, 2H, H-2), 1.16 (d, J = 6.0, 3H, H-6).

AF−18: elentheroside E[18]

13C NMR (151 MHz, Deuterium Oxide) δ 140.6 (C-1,1′), 155.4 (C-2,2′), 104.0 (C-3,3′,1′′,1′′′), 129.0 (C-4,4′), 105.4 (C-5,5′), 155.4 (C-6,6′), 88.4 (C-7,10), 54.5 (C-8,11), 72.9 (C-9,12), 73.7 (C-2′′,2′′′), 78.4 (C-3′′,3′′′), 70.7 (C-4′′,4′′′), 77.8 (C-5′′,5′′′), 61.4 (C-6′′,6′′′), 56.5 (C-2,6,2′,6′-OMe).

1H NMR (600 MHz, Deuterium Oxide) δ 6.63 (td, J = 22.1, 10.5 Hz, 4H, H-3, 5, 3′, 5′), 4.42 - 4.23 (m, 2H, H-1′′, 1′′′), 4.15 - 4.08 (m, 2H, H-4′′, H-4′′′), 3.79 - 3.61 (m, 2H, H-3′′, 3′′′), 3.63 - 3.46 (m, 4H, H-6′′, 6′′′), 3.54 (s, 4H, H-2′′, 5′′, 2′′′, 5′′′), 3.36 - 3.19 (m, 6H, H-7, 9, 10, 12), 3.19 (s, 2H, H-8, 11), 3.18 - 3.07 (m, 12H, -OCH3).

AF−19: methyl−*α*−D−glucopyranoside[19]

13C NMR (151 MHz, Deuterium Oxide) δ 100.6 (C-1), 73.1 (C-2), 75.6 (C-3), 72.0 (C-4), 74.7 (C-5), 60.6 (C-6), 57.0 (C-7).

1H NMR (600 MHz, Deuterium Oxide) δ 4.00 - 3.94 (m, 1H, H-1), 3.89 (s, 1H, H-6a), 3.89 - 3.74 (m, 1H, H-6b), 3.76 - 3.50 (m, 1H, H-3), 3.49 - 3.37 (m, 1H, H-5), 3.34 (s, 1H, H-2), 3.34 (s, 1H, H-4), 3.25 (s, 1H, H-7).

AF−20: burkholone[20]

13C NMR (151 MHz, Chloroform-*d*) δ 147.2 (C-2), 115.3 (C-3), 179.5 (C-4), 124.6 (C-5), 119.1 (C-6), 131.9 (C-7), 116.0 (C-8), 124.0 (C-4a), 138.6 (C-8a), 34.1 (C-1′), 124.0 (C-2′), 133.0 (C-3′), 32.0 (C-4′), 27.0 (C-5′), 31.5 (C-6′), 22.9 (C-7′), 14.2 (C-8′), 10.1 (C-9′).

1H NMR (600 MHz, Chloroform-*d*) δ 9.87 - 9.74 (brs, 1H, 1-NH), 7.92 (dd, 1H, H-5), 7.70 (d, 1H, H-7), 7.51 (dd, 1H, H-8), 7.25 (s, 1H, H-6), 5.35 (dt, 1H, H-3′), 5.06 (d, 1H, H-2′), 3.47 (s, 2H, H-1′) 2.32 (t, J = 7.5 Hz, 3H, H-9′), 1.64 - 1.59 (m, 2H, H-4′), 1.46 - 1.38 (m, 2H, H-5′), 1.41 (s, 2H, H-6′), 1.27 (s, 2H, H-7′), 0.90 - 0.77 (m, 3H, H-8′).

AF−21: gynostemoside C[21]

13C NMR (151 MHz, Methanol-*d*$_4$) δ 33.6 (C-1), 40.0 (C-2), 71.1 (C-3), 74.6 (C-4), 29.4 (C-5), 50.4 (C-6), 132.4 (C-7), 134.5 (C-8), 76.5 (C-9), 18.5 (C-10), 31.4 (C-11), 20.6 (C-12), 16.6 (C-13), 103.2 (C-1′), 76.4 (C-2′), 78.0 (C-3′), 70.2 (C-4′), 78.0 (C-5′), 61.5 (C-6′).

1H NMR (600 MHz, Methanol-*d*$_4$) δ 5.19 (dd, J = 22.0, 1.7 Hz, 1H, H-8), 5.16 (d, J = 1.8 Hz, 1H, H-7), 4.96 (s, 1H, H-1′), 4.46 (dd, J = 13.3, 7.8 Hz, 1H, H-9), 4.41 - 4.31 (m, 2H, H-6′), 4.21 (dd, J = 7.8, 1.5 Hz, 1H, H-3), 4.11 (dt, J = 11.4, 5.9 Hz, 2H, H-3′, 4′), 3.92 - 3.74 (d, 2H, H-4, 2′), 3.74 - 3.68 (m, 1H, H-5′), 2.41 (s, 2H, H-5, 6), 2.16 - 1.89 (m, J = 6.4 Hz, 2H, H-2), 1.70 (dd, J = 8.7, 6.0 Hz, 3H, H-10), 1.65 - 1.49 (m, 3H, H-12), 1.26 (d, 3H, H-13), 1.27 - 1.15 (m, 3H, H-11).

AF−22: 5,7−dihydroxy−2−pentadecylchromen−4−one[22]

13C NMR (151 MHz, Methanol-*d*$_4$) δ 164.2 (C-2), 106.8 (C-3), 181.0 (C-4), 161.4 (C-5), 97.9 (C-6), 159.1 (C-7), 93.1 (C-8), 103.4 (C-4a), 157.2 (C-8a), 35.2 (C-1′), 31.7 (C-2′), 29.5 (C-3′,4′), 29.2 (C-5′,6′), 29.1 (C-7′,8′), 29.0 (C-9′,10′), 26.7 (C-11′,12′), 22.4 (C-13′,14′), 13.1 (C-15′).

1H NMR (600 MHz, Methanol-*d*$_4$) δ 8.09 - 8.04 (m, 1H, 5-OH), 6.91 - 6.86 (m, 1H, 7-OH), 6.37 (d, J = 2.0 Hz, 1H, H-8), 6.16 (d, J = 2.1 Hz, 1H, H-3), 5.36 - 5.31 (m, 1H, H-6), 2.01 - 1.97 (m, 2H, H-1′), 1.62 - 1.08 (m, 26H, H- 2′ ~ 14′), 0.92 - 0.81 (m, 3H, H-15′).

AF−23: anisodamine[23]

13C NMR (151 MHz, Deuterium Oxide) δ 63.8 (C-1), 36.3 (C-2), 68.9 (C-3), 36.3 (C-4), 69.3 (C-5), 69.7 (C-6), 36.3 (C-7), 42.9 (C-8), 174.0 (C-9), 56.0 (C-10), 63.8 (C-11), 135.0 (C-12), 129.1 (C-13), 129.3 (C-14), 127.6 (C-15), 129.3 (C-16), 129.1 (C-17).

1H NMR (600 MHz, Deuterium Oxide) δ 7.25 - 7.17 (m, 2H, H-14,16), 7.20 - 7.14 (m, 1H, H-15), 7.14 - 7.08 (m, 2H, H-13,17), 3.85 - 3.79 (m, 1H, H-11*α*), 3.81 - 3.75 (m, 1H, H-11β), 3.68 (s, 1H, H-3), 3.74 - 3.60 (m, 1H, H-6), 3.62 - 3.43 (m, 1H, H-10), 3.11 - 3.05 (m, 1H, H-1), 3.01 (d, J = 7.5 Hz, 2H, H-5), 2.92 (s, 3H, H-8), 2.90 (dd, J = 14.5, 7.9 Hz, 2H, H-4), 1.55 (s, 2H, H-7), 1.50 (dd, J = 12.0, 6.9 Hz, 2H, H-2).

AF−24: onopornoid C[24]

13C NMR (151 MHz, Chloroform-*d*) δ 148.5 (C-1), 105.3 (C-2), 105.5 (C-3), 145.8 (C-4), 50.3 (C-5), 86.9 (C-6), 52.7 (C-7), 71.9 (C-8), 41.7 (C-9), 38.6 (C-10), 41.1 (C-11), 180.4 (C-12), 55.3 (C-13), 18.0 (C-14), 67.3 (C-15), 175.0 (C-1′), 72.3 (C-2′), 29.6 (C-3′), 24.6 (C-4′), 56.1 (C-5′), 167.8 (C-1′′), 131.5 (C-2′′), 139.6 (C-3′′), 14.2 (C-4′′), 54.9 (C-5′′).

1H NMR (600 MHz, Chloroform-*d*) δ 7.49 (s, 1H, H-3′′), 6.54 (s, 1H, H-1), 5.28 (s, 2H, H-3), 5.14 (s, 1H, H-8), 5.06 (dd, J = 9.9, 5.2 Hz, 2H, H-2), 4.52 - 4.47 (m, 1H, H-6), 4.43 (s, 2H, H-5′′), 4.20 - 4.14 (m, 1H, H-15), 3.87 (s, 1H, H-2′), 3.77 (s, 2H, H-5′), 3.62 (dt, J = 11.6, 7.7 Hz, 2H, H-13), 3.46 - 3.39 (m, 1H, H-11), 2.86 - 2.80 (m, 1H, H-5), 2.70 (s, 1H, H-7), 2.36 (s, 2H, H-3′), 2.08 (d, J = 14.0 Hz, 2H, H-4′), 1.99 - 1.94 (m, 3H, H-4′′), 1.94 - 1.88 (m, 2H, H-9), 1.22 (s, 3H, H-14).

AF−25: 3−methylspongia−3,12−dien−16−one[25]

13C NMR (151 MHz, Chloroform-*d*) δ 32.0 (C-1), 29.8 (C-2), 126.9 (C-3), 128.6 (C-4), 50.6 (C-5), 22.8 (C-6), 41.7 (C-7), 34.5 (C-8), 53.0 (C-9), 37.1 (C-10), 24.9 (C-11), 138.3 (C-12), 129.2 (C-13), 52.3 (C-14), 69.6 (C-15), 173.0 (C-16), 14.2 (C-17), 11.4 (C-18), 18.0 (C-19), 21.8 (C-20).

1H NMR (600 MHz, Chloroform-*d*) δ 6.98 (s, 1H, H-12), 4.74 (d, 2H, H-15), 3.03 - 2.98 (m, 1H, H-14), 2.61 (s, 1H, H-11a), 2.31 - 2.24 (m, 1H, H-11b), 1.99 (s, 2H, H-2), 1.92 (d, 1H, H-5), 1.70 (s, 2H, H-6), 1.65 (s, 2H, H-7), 1.60 (s, 3H, H-19), 1.59 (m, 3H, H-20), 1.48 (s, 1H, H-9), 1.24 (s, 2H, H-1), 0.98 - 0.84 (m, 3H, H-17), 0.83 (s, 3H, H-18).

AF−26: methyl 2,3,4,6−tetra−O−methyl−*α*−D−mannopyranosyl−(1→ 4) −6−O−acetyl−2,3−di−O−methyl−*α*−D−glucopyranoside[26]

13C NMR (151 MHz, Methanol-*d*4) δ 100.9 (C-1), 81.5 (C-2), 83.3 (C-3), 74.4 (C-4), 97.8 (C-5), 63.6 (C-6), 59.1 (C-7), 59.1 (C-8), 59.5 (C-9), 61.5 (C-10), 107.9 (C-1′), 81.1 (C-2′), 77.6 (C-3′,4′), 72.8 (C-5′), 71.5 (C-6′), 57.4 (C-7′), 59.1 (C-8′,9′), 179.9 (C-10′), 21.2 (C-11′).

1H NMR (600 MHz, Methanol-*d*4) δ 5.47 (s, 1H, H-1′), 4.49 (d, 1H, H-1), 4.36 (s, 1H, H-6′a), 4.34 (s, 1H, H-6′b), 4.21- 4.19 (dd, 1H, H-5′), 4.01 (d, J = 4.2 Hz, 1H, H-5), 3.87 (dd, J = 6.5, 4.2 Hz, 4H, H-2, 3, 2′, 3′), 3.82 (ddd, J = 6.5, 5.0, 3.0 Hz, 2H, H-4, 4′), 3.73 (dd, J = 11.9, 3.0 Hz, 1H, H-6b), 3.68 (d, J = 12.0 Hz, 1H, H-6a), 3.64 - 3.28 (m, OMe), 1.85 (s, 3H, H-11′).

AF−27: β1−chaconine[27]

13C NMR (151 MHz, DMSO-*d*6) δ 37.3 (C-1), 29.5 (C-2), 76.5 (C-3), 38.2 (C-4), 140.7 (C-5), 122.2 (C-6), 31.5 (C-7), 30.3 (C-8), 50.1 (C-9), 36.9 (C-10), 56.3 (C-11), 41.6 (C-12), 38.2 (C-13), 21.0 (C-14), 32.1 (C-15), 73.9 (C-16), 66.4 (C-17), 15.2 (C-18), 19.5 (C-19), 62.3 (C-20), 31.5 (C-21), 29.0 (C-22), 28.9 (C-23), 81.4 (C-24), 36.9 (C-25), 17.6 (C-26), 16.5 (C-27), 98.9 (C-1′), 81.0 (C-2′), 76.8 (C-3′), 70.4 (C-4′), 77.6 (C-5′), 62.3 (C-6′), 100.3 (C-1′′), 72.3 (C-2′′), 71.0 (C-3′′), 73.5 (C-4′′), 68.5 (C-5′′), 18.3 (C-6′′).

1H NMR (600 MHz, DMSO-*d*6) δ 5.29 (q, J = 2.5 Hz, 1H, H-1′), 5.02 (d, J = 1.6 Hz, 1H, H-1′′), 4.63 (s, 1H, H-6), 4.41 (d, J = 7.9 Hz, 1H, H-2′′), 4.24 (dd, J = 8.3, 6.5 Hz, 1H, H-3′), 4.19 (d, J = 7.9 Hz, 2H, H-3′′, 5′′), 3.96 (dd, J = 9.5, 6.2 Hz, 1H, H-2′), 3.68 (d, J = 11.4 Hz, 1H, H-4′), 3.66 - 3.62 (m, 1H, H-5′), 3.57 (dd, J = 3.3, 1.7 Hz, 1H, H-4′′), 3.55 - 3.50 (m, 3H, H-6′′), 3.50 - 3.40 (m, 2H, H-6′), 3.39 - 3.33 (m, 1H, H-3), 3.28 - 3.23 (m, 2H, H-20), 3.23 - 3.08 (m, 4H, H-4, 7), 3.00 (t, J = 9.3 Hz, 1H, H-16), 2.93 (dd, J = 9.1, 7.9 Hz, 1H, H-24), 1.88 (s, 2H, H-15), 1.90 - 1.82 (m, 1H, H-21), 1.81 - 1.73 (m, 6H, H-13, 22, 23), 1.75 (s, 3H, H-2, 25), 1.67 - 1.42 (m, 2H, H-14), 1.41 - 1.32 (m, 3H, H-1, 8), 1.31 - 1.22 (m, 1H, H-9), 1.17 - 1.09 (m, 1H, H-17), 1.11 - 1.00 (m, 4H, H-11, 19), 0.92 (s, 3H, H-27), 0.86 (d, J = 7.0 Hz, 3H, H-26), 0.69 (t, J = 3.2 Hz, 3H, H-18).

AF−28: pestalafuranone E[28]

13C NMR (151 MHz, Methanol-*d*4) δ 173.3 (C-2), 127.2 (C-3), 159.3 (C-4), 74.2 (C-5), 24.8 (C-6), 22.2 (C-7), 13.2 (C-8), 29.4 (C-9), 57.0 (C-10), 54.3 (C-11), 17.6 (C-12).

1H NMR (600 MHz, Methanol-*d*4) δ 4.60 (t, J = 6.1 Hz, 2H, H-5), 3.75 (s, 2H, H-9), 3.64 (dd, J = 9.8, 4.1 Hz, 1H, H-11), 3.62 (s, 1H, H-10), 3.56 (s, 1H, H-6a), 3.57 - 3.48 (m, 1H, H-6b), 1.29 (s, 2H, H-7), 0.83 (s, 6H, H-8, 12).

AF−29: nortetillapyrone[29]

13C NMR (151 MHz, Methanol-*d*4) δ 151.3 (C-2), 101.6 (C-3), 141.6 (C-4), 102.6 (C-5), 165.3 (C-6), 87.4 (C-7), 39.8 (C-8), 70.9 (C-9), 89.4 (C-10), 61.6 (C-11).

1H NMR (600 MHz, Methanol-*d*4) δ 7.95 (dd, J = 20.4, 8.1 Hz, 1H, H-4), 6.24 (t, J = 6.8 Hz, 1H, H-7), 5.87 (d, J = 4.6 Hz, 1H, H-3), 4.38 (tt, J = 7.2, 3.4 Hz, 1H, H-9), 3.75 - 3.69 (m, 1H, H-10), 3.51 (dd, J = 11.3, 6.1 Hz, 2H, H-11), 2.36 - 2.28 (m, 2H, H-8).

AF−30: tricosanol[30]

13C NMR (151 MHz, Methanol-*d*4) δ 67.1 (C-1), 33.6 (C-2), 31.7 (C-3), 29.4 (C-4~16), 29.1 (C-17~20), 24.8 (C-21), 22.4 (C-22), 13.0 (C-23).

1H NMR (600 MHz, Methanol-*d*4) δ 3.71 - 3.59 (m, 2H, H-1), 1.27 - 1.26 (s, 42H, H-2~22), 0.87 (s, 3H, H-23).

AF−31: pentacosane[31]

13C NMR (151 MHz, Methanol-*d*4) δ 13.2 (C-1,25), 22.4 (C-2,24), 31.7 (C-3,23), 29.1 (C-4,22), 29.4 (C-5,21).

1H NMR (600 MHz, Methanol-*d*4) δ 1.31 - 1.26 (m, 46H, H-2~24), 0.90 – 0.85 (m, 6H, H-1, 25).

AF−32: pinellactam[32]

13C NMR (151 MHz, Methanol-*d*4) δ 59.6 (C-2), 24.7 (C-3), 29.0 (C-4), 179.7 (C-5), 51.5 (C-6), 173.1 (C-7), 13.1 (C-8), 55.6 (C-9), 175.2 (C-10), 55.6 (C-11).

1H NMR (600 MHz, Methanol-*d*4) δ 4.30 - 4.25 (m, 2H, H-2, 6), 3.73 (s, 6H, H-9, 11), 2.46 - 2.40 (m, 2H, H-4), 2.38 - 2.24 (m, 2H, H-3) 1.31 (s, 1H, H-8).

AF−33: kaempferol−3−O−β−D−4′′, 6′′ −di−(E) −p−coumaroyl glucoside[33]

13C NMR (151 MHz, Methanol-*d*4) δ 157.8 (C-2), 134.0 (C-3), 178.0 (C-4), 157.1 (C-5), 98.6 (C-6), 166.0 (C-7), 93.5 (C-8), 104.4 (C-4a), 164.6 (C-8a), 121.4 (C-1′), 132.6 (C-2′), 117.0 (C-3′), 159.2 (C-4′), 117.0 (C-5′), 132.6 (C-6′), 102.6 (C-1′′), 72.1 (C-2′′), 70.8 (C-3′′), 75.2 (C-4′′), 74.6 (C-5′′), 61.0 (C-6′′), 168.2 (C-1′′′), 114.5 (C-2′′′), 146.0 (C-3′′′), 129.3 (C-4′′′), 131.0 (C-5′′′), 115.4 (C-6′′′), 160.3 (C-7′′′), 115.5 (C-8′′′), 131.0 (C-9′′′), 167.2 (C-1′′′′), 113.4 (C-2′′′′), 144.8 (C-3′′′′), 127.1 (C-4′′′′), 130.0 (C-5′′′′), 114.8 (C-6′′′′), 161.7 (C-7′′′′), 114.8 (C-8′′′′), 130.0 (C-9′′′′).

1H NMR (600 MHz, Methanol-*d*4) δ 8.03 (ddd, J = 8.8, 4.2, 2.0 Hz, 2H, H-2′, 6′), 7.69 - 7.59 (m, 1H, H-3′′′), 7.47 - 7.39 (m, 2H, H-5′′′, 9′′′), 7.39 - 7.35 (m, 1H, H-3′′′′), 7.22 - 7.17 (m, 2H, H-5′′′′, 9′′′′), 6.91 - 6.83 (m, 2H, H-3′, 5′), 6.81 - 6.69 (m, 4H, H-6′′′, 8′′′, 6′′′′, 8′′′′), 6.42 - 6.34 (m, 1H, H-2′′′), 6.17 (q, J = 1.7 Hz, 3H, H-6, 8, 2′′′′), 5.33 (d, 1H, H-1′′, 4′′), 4.07 (d, 2H, H-6′′), 3.72 - 3.60 (m, 1H, H-2′′), 3.58 - 3.51 (m, 2H, H-3′′, 5′′).

AF−34: kaempferol 3−O−β−D−glucopyranosyl−(1→ 2) −β−D−glucopyranosyl−(1→ 6) −β−D− glucopyranodide[34]

13C NMR (151 MHz, Methanol-*d*4) δ 157.0 (C-2), 134.1 (C-3), 178.1 (C-4), 161.6 (C-5), 98.6 (C-6), 164.5 (C-7), 93.5 (C-8), 104.4 (C-4a), 157.7 (C-8a), 121.4 (C-1′), 131.0 (C-2′), 114.8 (C-3′), 160.2 (C-4′), 114.8 (C-5′), 131.0 (C-6′), 101.4 (C-1′′), 74.4 (C-2′′), 75.8 (C-3′′), 70.2 (C-4′′), 75.8 (C-5′′), 69.3 (C-6′′), 102.9 (C-1′′′), 82.1 (C-2′′′), 75.8 (C-3′′′), 70.1 (C-4′′′), 76.7 (C-5′′′), 61.3 (C-6′′′), 103.9 (C-1′′′′), 74.4 (C-2′′′′), 77.0 (C-3′′′′), 70.0 (C-4′′′′), 76.7 (C-5′′′′), 63.0 (C-6′′′′).

1H NMR (600 MHz, Methanol-*d*4) δ 8.01 (d, J = 6.9 Hz, 1H, H-2′), 8.00 (s, 1H, H-6′), 6.88 – 6.82 (m, 2H, H-3′, 5′), 6.32 (dd, J = 4.8, 2.1 Hz, 1H, H-8), 6.14 (d, J = 2.1 Hz, 1H, H-6), 5.23 – 5.18 (m, 1H, H-1′′), 4.17 – 4.07 (m, 1H, H-1′′′), 3.95 – 3.87 (m, 1H, H-1′′′′), 3.85 – 3.79 (m, 1H, H-4′′′), 3.77 – 3.39 (m, 12H, H-2′′, 5′′, 6′′, 3′′′, 5′′′, 6′′′, 4′′′′, 5′′′′,6′′′′), 3.32 – 3.23 (m, 4H, H-3′′, 4′′, 2′′′, 3′′′′), 3.20 (ddd, J = 9.8, 5.4, 2.4 Hz, 1H, H-2′′′′).

AF−35: *α*−D−fructofuranose[35]

13C NMR (151 MHz, Methanol-*d*4) δ 61.5 (C-1), 107.9 (C-2), 81.1 (C-3), 77.5 (C-4), 83.3 (C-5), 59.1 (C-6).

1H NMR (600 MHz, Methanol-*d*4) δ 4.00 (d, J = 4.2 Hz, 1H, H-3), 3.87 (dd, J = 6.5, 4.2 Hz, 1H, H-4), 3.81 (ddd, J = 6.5, 5.0, 3.0 Hz, 1H, H-5), 3.73 (dd, J = 11.9, 3.0 Hz, 1H, H-6a), 3.68 (d, J = 12.0 Hz, 1H, H-6b), 3.64 - 3.58 (m, 2H, H-1).

AF−36: isorhamnetin 3−O−β−D−(6−acetyl) −galactopyranoside[36]

13C NMR (151 MHz, Methanol-*d*4) δ 159.6 (C-2), 132.1 (C-3), 176.5 (C-4), 161.6 (C-5), 98.4 (C-6), 164.7 (C-7), 93.6 (C-8), 103.6 (C-4a), 157.3 (C-8a), 122.3 (C-1′), 113.7 (C-2′), 148.4 (C-3′), 147.1 (C-4′), 115.4 (C-5′), 122.8 (C-6′), 55.5 (C-7′), 103.6 (C-1′′), 72.4 (C-2′′), 73.6 (C-3′′), 67.8 (C-4′′), 73.6 (C-5′′), 62.0 (C-6′′), 169.2 (C-7′′), 20.7 (C-8′′).

1H NMR (600 MHz, Methanol-*d*4) δ 7.85 (d, J = 8.8 Hz, 1H, H-2′), 7.02 (d, J = 8.5 Hz, 1H, H-6′), 6.79 (d, J = 2.1 Hz, 1H, H-3′), 6.80 – 6.74 (m, 1H, H-8), 6.69 (d, J = 8.4 Hz, 1H, H-

6), 6.36 (d, J = 2.1 Hz, 1H, H-1''), 6.15 (d, J = 2.0 Hz, 3H, H-2'', 3'', 4''), 4.40 (dd, J = 10.2, 4.1 Hz, 2H, H-6''), 3.90 (s, 1H, H-5''), 3.87 – 3.84 (m, 3H, H-7'), 1.36 (s, 3H, H-8'').

AF−37: rhamnocitrin[37]

13C NMR (151 MHz, Methanol-*d*4) δ 146.8 (C-2), 135.8 (C-3), 176.0 (C-4), 156.9 (C-5), 97.9 (C-6), 164.2 (C-7), 93.1 (C-8), 103.2 (C-4a), 161.1 (C-8a), 55.2 (C-9), 122.4 (C-1'), 129.4 (C-2'), 115.0 (C-3'), 159.2 (C-4'), 115.0 (C-5'), 129.4 (C-6').

1H NMR (600 MHz, Methanol-*d*4) δ 8.08 - 8.03 (m, 2H, H-3', 5'), 6.91 - 6.86 (m, 2H, H-2', 6'), 6.37 (d, J = 2.1 Hz, 1H, H-8), 6.16 (d, J = 2.0 Hz, 1H, H-6), 3.90 (d, 3H, H-9).

AF−38: macasiamenol B[38]

13C NMR (151 MHz, Methanol-*d*4) δ 145.3 (C-2), 140.7 (C-3), 176.0 (C-4), 161.2 (C-5), 97.9 (C-6), 164.2 (C-7), 93.1 (C-8), 103.2 (C-4a), 157.0 (C-8a), 122.4 (C-1'), 129.7 (C-2'), 131.6 (C-3'), 159.2 (C-4'), 114.9 (C-5'), 129.3 (C-6'), 29.4 (C-7'), 121.9 (C-8'), 135.8 (C-9'), 17.3 (C-10'), 24.5 (C-11'), 55.0 (C-12').

1H NMR (600 MHz, Methanol-*d*4) δ 8.06 (d, J = 8.9 Hz, 1H, H-6'), 7.87 - 7.83 (m, 1H, H-2'), 7.09 (d, J = 2.0 Hz, 1H, H-5'), 6.72 - 6.66 (m, 1H, H-8), 6.37 (d, J = 2.1 Hz, 1H, H-6), 5.36 - 5.28 (m, 1H, H-8'), 3.46 - 3.41 (m, 2H, H-7'), 3.28 (m, J = 1.6 Hz, 3H, H-12'), 1.25 (s, 6H, H-10', 11').

AF−39: afzelin[39]

13C NMR (151 MHz, Methanol-*d*4) δ 157.6 (C-2), 134.1 (C-3), 178.0 (C-4), 161.8 (C-5), 98.5 (C-6), 165.0 (C-7), 93.4 (C-8), 104.4 (C-4a), 157.2 (C-8a), 121.4 (C-1'), 131.0 (C-2'), 114.7 (C-3'), 160.2 (C-4'), 114.7 (C-5'), 131.0 (C-6'), 102.8 (C-1''), 70.0 (C-2''), 74.4 (C-3''), 77.0 (C-4''), 76.7 (C-5''), 17.6 (C-6'').

1H NMR (600 MHz, Methanol-*d*4) δ 8.05 - 7.99 (m, 2H, H-2', 6'), 6.89 - 6.82 (m, 2H, H-3', 5'), 6.34 (s, 1H, H-8), 5.38 (d, 1H, H-6), 5.22 (s, 1H, H-1''), 3.67 (dd, J = 11.9, 2.4 Hz, 2H, H-2'', 5''), 3.51 (dd, J = 11.9, 5.5 Hz, 1H, H-3''), 3.40 (s, 1H, H-4''), 1.20 (m, 3H, H-6'').

AF−40: tamarixin[40]

13C NMR (151 MHz, Methanol-*d*4) δ 160.0 (C-2), 134.0 (C-3), 177.8 (C-4), 161.8 (C-5), 102.1 (C-6), 165.1 (C-7), 98.8 (C-8), 102.6 (C-4a), 157.6 (C-8a), 122.3 (C-1'), 112.7 (C-2'), 157.0 (C-3'), 149.2 (C-4'), 114.6 (C-5'), 130.8 (C-6'), 55.3 (C-7'), 104.1 (C-1''), 74.2 (C-2''), 76.4 (C-3''), 70.0 (C-4''), 76.8 (C-5''), 61.0 (C-6'').

1H NMR (600 MHz, Methanol-*d*4) δ 8.04 - 7.99 (s, 1H, H-2'), 7.87 (d, J = 2.0 Hz, 2H, H-6', 5'), 6.87 (t, J = 8.4 Hz, 1H, H-8), 6.34 (s, 1H, H-6), 6.16 (s, 1H, H-1''), 3.91- 3.70 (m, 6H, glucose protons), 3.66 (dd, J = 12.0, 2.3 Hz, 3H, H-7').

References

1. Li, M.X.; Xie, J.; Bai, X.; Du, Z.Z. Anti-aging potential, anti-tyrosinase and antibacterial activities of extracts and compounds isolated from Rosa chinensis cv. 'JinBian.' Ind. Crops Prod. 2021, 159, 113059.
2. Zhang, W.Z.; Li, X.L.; Shi, L.G.; Wang, J.L.; Zhao, M.; Zhao, D.F.; Zhang, S.J. Sesquiterpene lactones from Ixeris sonchifolia (Bge.) Hance II. J. Asian Nat. Prod. Res. 2008, 10, 1087–1091.
3. Nagashima, F.; Suzuki, M.; Asakawa, Y. seco-cuparane-type sesquiterpenoid from the Japanese liverwort Jungermannia infusca. Phytochemistry 2001, 56, 807–810.
4. Parmar, V.S.; Bisht, K.S.; Malhotra, A.; Jha, A.; Errington, W.; Howarth, O.W.; Tyagi, O.D.; Stein, P.C.; Jensen, S.; Boll, P.M.; et al. A benzoic acid ester from uvaria narum. Phytochemistry 1995, 38, 951–955.
5. Erb, B.; Borschberg, H.J.; Arigoni, D. The structure of laricinolic acid and its biomimetic transformation into officinalic acid. J. Chem. Soc. Perkin Trans. 1 2000, 31, 2307–2309.
6. Samoylenko, V.; Rahman, M.M.; Tekwani, B.L.; Tripathi, L.M.; Wang, Y.H.; Khan, S.I.; Khan, I.A.; Miller, L.S.; Joshi, V.C.; Muhammad, I. Banisteriopsis caapi, a unique combination of MAO inhibitory and antioxidative constituents for the activities relevant to neurodegenerative disorders and Parkinson's disease. J. Ethnopharmacol. 2010, 127, 357–367.
7. Paridhavi, M.; Agrawal, S.S. Isolation and characterization of flowers of Rosa damascena. Asian J. Chem. 2007, 19, 2751–2756.
8. Liu, C. Da; Chen, J.; Wang, J.H. A novel kaempferol triglycoside from flower buds of Panax quinquefolium. Chem. Nat. Compd. 2009, 45, 808–810.
9. Rezende, F.M.; Ferreira, M.J.P.; Clausen, M.H.; Rossi, M.; Furlan, C.M. Acylated flavonoid glycosides are the main pigments that determine the flower colour of the brazilian native tree tibouchina pulchra (Cham.) Cogn. Molecules 2019, 24, 718.
10. Tewari, A.; Bhakuni, R.S. Terpenoid and lipid constituents from Artemisia annua. Indian J. Chem. - Sect. B Org. Med. Chem. 2003, 42, 1782–1785.

11. Gou, S.H.; Liu, J.; He, M.; Qiang, Y.; Ni, J.M. Quantification and bio-assay of α-glucosidase inhibitors from the roots of Glycyrrhiza uralensis Fisch. Nat. Prod. Res. 2016, 30, 2130–2134.
12. Wang, C.Z.; Yu, D.Q. Lignan and acetylenic glycosides from Aster auriculatus. Phytochemistry 1998, 48, 711–717.
13. Hyun, S.K.; Jung, H.A.; Min, B.S.; Jung, J.H.; Choi, J.S. Isolation of phenolics, nucleosides, saccharides and an alkaloid from the root of Aralia cordata. Nat. Prod. Sci. 2010, 16, 20–25.
14. Nycz, J.E.; Malecki, G.; Morag, M.; Nowak, G.; Ponikiewski, L.; Kusz, J.; Switlicka, A. Arbutin: Isolation, X-ray structure and computional studies. J. Mol. Struct. 2010, 980, 13–17.
15. Wei-li, Y.; Jun, T.; Liu-sheng, D. Chemical Constituents of Gymnotheca involucrata Pei. China J. Chinese Mater. Medica 2001, 26, 43–44.
16. Zhou, Y.Z.; Ma, H.Y.; Chen, H.; Qiao, L.; Yao, Y.; Cao, J.Q.; Pei, Y.H. New acetylenic glucosides from Carthamus tinctorius. Chem. Pharm. Bull. 2006, 54, 1455–1456.
17. Ding, Z.; Liu, Y.; Ruan, J.; Yang, S.; Yu, H.; Chen, M.; Zhang, Y.; Wang, T. Bioactive constituents from the whole plants of gentianella acuta (Michx.) Hulten. Molecules 2017, 22, 1–13.
18. Chun-xia, X.; Yan, X.; Cai-xia, D.; Chun-mao, Y.; Lie-jun, H.; Wei, C.; Xiao-jiang, H. Chemical Constituents from Water-soluble Parts of Baphicacanthus cusia. J. Chinese Med. Mater. 2020, 43, 596–601.
19. Abou-Hussein, D.R.; Badr, J.M.; Youssef, D.T.A. Dragmacidoside: A new nucleoside from the red sea sponge dragmacidon coccinea. Nat. Prod. Res. 2014, 28, 1134–1141.
20. Mori, T.; Yamashita, T.; Furihata, K.; Nagai, K.; Suzuki, K.I.; Hayakawa, Y.; Shin-ya, K. Burkholone, a new cytotoxic antibiotic against IGF-I dependent cells from Burkholderia sp. J. Antibiot. (Tokyo). 2007, 60, 713–716.
21. Zhang, Z.; Zhang, W.; Ji, Y.P.; Zhao, Y.; Wang, C.G.; Hu, J.F. Gynostemosides A-E, megastigmane glycosides from Gynostemma pentaphyllum. Phytochemistry 2010, 71, 693–700.
22. Rovirosa, J.; Sepulveda, M.; Quezada, E.; San-Martin, A. Isoepitaondiol, a diterpenoid of Stypopodium flabelliforme and the insecticidal activity of stypotriol, epitaondiol and derivatives. Phytochemistry 1992, 31, 2679–2681.
23. Zang, E.H.; Chen, Z.W.; Zhang, C.H.; Li, M.H. Chemical constituents of Physochlaina physaloides (L.) G. Don (Solanaceae). Biochem. Syst. Ecol. 2021, 98, 104332.
24. Sugimoto, S.; Yamano, Y.; Desoukey, S.Y.; Katakawa, K.; Wanas, A.S.; Otsuka, H.; Matsunami, K. Isolation of Sesquiterpene-Amino Acid Conjugates, Onopornoids A-D, and a Flavonoid Glucoside from Onopordum alexandrinum. J. Nat. Prod. 2019, 82, 1471–1477.
25. Costa, M.; Fernández, R.; Pérez, M.; Thorsteinsdottir, M. Two new spongian diterpene analogues isolated from the marine sponge Acanthodendrilla sp. Nat. Prod. Res. 2020, 34, 1053–1060.
26. León, E.I.; Martín, A.; Peréz-Martín, I.; Quintanal, L.M.; Suárez, E. Hydrogen atom transfer experiments provide chemical evidence for the conformational differences between C-and O-disaccharides. European J. Org. Chem. 2010, 2010, 5248–5262.
27. Kitajima, J.; Komori, T.; Kawasaki, T.; Schulten, H. rolf Basic steroid saponins from aerial parts of Fritillaria Thunbergii. Phytochemistry 1982, 21, 187–192.
28. Liu, H.; Liu, S.; Guo, L.; Zhang, Y.; Cui, L.; Ding, G. New furanones from the plant endophytic fungus Pestalotiopsis besseyi. Molecules 2012, 17, 14015–14021.
29. El-Gamal, A.A.; Al-Massarani, S.M.; Shaala, L.A.; Alahdald, A.M.; Al-Said, M.S.; Ashour, A.E.; Kumar, A.; Abdel-Kader, M.S.; Abdel-Mageed, W.M.; Youssef, D.T.A. Cytotoxic compounds from the Saudi red sea sponge xestospongia testudinaria. Mar. Drugs 2016, 14, 1–9.
30. Sun, F.; Zhang, L.; Tian, J.K.; Cheng, J.Y.; Xiao, P.G. Studies on Chemical Constituents of Clematis terniflora. Chinese Pharm. J. 2007, 42, 102–103.
31. Huang, X.A.; Yang, R.Z. A new hydroquinone diglucoside from Lysimachia fordiana. Chem. Nat. Compd. 2004, 40, 457–459.
32. Gao, Q.; Cheng, Y. A New Lactam from Pinellia ternata. Nat. Prod. Res. Dev. 2015, 27, 1693–1696.
33. Yang, C.P.; Shie, P.H.; Huang, G.J.; Chien, S.C.; Kuo, Y.H. New anti-inflammatory flavonol glycosides from lindera akoensis hayata. Molecules 2019, 24, 1–11.
34. Lin, Q.G.; Jie, O.Y.; Yun, K. De; Shan, D.S. Studies on Chemical Condtituents of Primula maximowiczii Regel II. Chinese Pharm. J. 2008, 43, 1300–1304.
35. Zhang, Z.; Wang, D.; Zhao, Y.; Gao, H.; Hu, Y.H.; Hu, J.F. Fructose-derived carbohydrates from Alisma orientalis. Nat. Prod. Res. 2009, 23, 1013–1020.
36. Gudej, J.; Nazaruk, J. Flavonol glycosides from the flowers of Bellis perennis. Fitoterapia 2001, 72, 839–840.
37. Johnst, P.I.M.; Barrientos, R.E.; Simirgiotis, M.J.; Palacios, J. Characterization of Polyphenol Compounds from Endothelium-Dependent Vascular Relaxation Effect in Rat Aorta. Molecules 2020, 25, 3105.
38. Pailee, P.; Sangpetsiripan, S.; Mahidol, C.; Ruchirawat, S.; Prachyawarakorn, V. Cytotoxic and cancer chemopreventive properties of prenylated stilbenoids from Macaranga siamensis. Tetrahedron 2015, 71, 5562–5571.
39. Duan, Y.; Hu, Y.; Yang, W.; Xiong, Y.; Du, C.; Yuan, C.; Hao, X.; Gu, W. Study on chemical constituents and α-glucosidase inhibitory activity of Cyclocarya paliurus in Guizhou province. Nat. Prod. Res. Dev. 2019, 31, 940–945.
40. Kavtaradze, N.; Alaniya, M.; Masullo, M.; Cerulli, A.; Piacente, S. New Flavone Glycosides from Astragalus tanae Endemic to Georgia. Chem. Nat. Compd. 2020, 56, 70–74.
